# Methodological Frameworks for Computational Electrocatalysis: From Theory to Practice

**DOI:** 10.1002/smtd.202501542

**Published:** 2026-02-15

**Authors:** Michele Re Fiorentin, Michele G. Bianchi, Magnus A. H. Christiansen, Anna Ciotti, Francesca Risplendi, Wei Wang, Elvar Ö. Jónsson, Hannes Jónsson, Giancarlo Cicero

**Affiliations:** ^1^ Department of Applied Science and Technology Politecnico di Torino Torino Italy; ^2^ Science Institute and Faculty of Physical Sciences, University of Iceland Reykjavik Iceland

**Keywords:** computational methods, density functional theory, electrochemical interface modeling, machine learning in atomistic simulations

## Abstract

Modeling electrocatalytic reactions at solid–liquid interfaces requires capturing both the quantum‐mechanical processes at the electrode surface and the complex response of the surrounding electrochemical environment. This review examines the main theoretical frameworks and computational techniques used to describe such systems, focusing on first‐principles approaches based on density functional theory (DFT). Key aspects include the treatment of reaction thermodynamics, electrode bias, solvation effects, electrolyte screening, and reaction kinetics. A broad range of methods is discussed, from thermochemical models, such as the computational hydrogen electrode, to potential‐dependent formulations based on grand‐canonical DFT and explicit calculation of kinetic barriers. The review also highlights recent machine‐learning approaches for catalyst screening and the growing use of machine‐learning‐based force fields, which promise to enable efficient simulations of complex electrochemical environments over extended time and length scales with near‐first‐principles accuracy. The aim is not only to present the state of the art, but also to clarify the physical assumptions and approximations underlying each approach. The influence of modeling choices on reliability and computational cost is examined in detail. Alongside theoretical aspects, practical considerations are emphasized to support researchers in selecting appropriate methods and designing simulations that are both physically meaningful and computationally tractable.

## Graphical abstract

Computational modeling is widely used to investigate electrocatalytic reactions, yet accurately describing electrochemical interfaces remains challenging. This review outlines theoretical and computational strategies, based on density functional theory, to model reaction thermodynamics, solvation effects, applied bias, and kinetics. Emphasis is placed on methodological assumptions, implementation details, strengths and limitations of each approach, including recent machine‐learning developments.

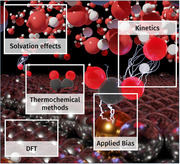

## 1 Introduction

Electrocatalytic processes are of central scientific importance due to their key role in enabling sustainable energy conversion and storage technologies. Electrochemical devices such as fuel cells and electrolyzers allow for either the generation of electricity from chemical fuels or the conversion of renewable electricity into storable chemical products. Key reactions such as the hydrogen evolution reaction, the oxygen evolution reaction, and the electrochemical reduction of ${\mathrm{CO}}_{2}$ and $N_{2}$ are fundamental to technologies for green hydrogen production, carbon‐neutral fuels, and sustainable fertilizer synthesis [1, 2, 3]. In all such applications, the efficiency, selectivity, and durability of electrocatalysts are critical factors. Understanding and improving these processes at the molecular level requires detailed insights into the solid–liquid interface where electrocatalysis occurs. In this context, computational modeling has become an indispensable tool, not only to interpret experimental results, but to uncover reaction mechanisms and guide the rational design of new catalytic materials [4].

Theoretical modeling of electrocatalytic systems is inherently challenging because it requires combining quantum‐mechanical accuracy with the statistical complexity of a fluctuating liquid environment under electric bias. Central to these processes is the electrified solid–liquid interface, where bond making and breaking at the atomic scale occur in the presence of long‐range electrostatic fields, solvent polarization, and mobile ionic species. Capturing this interface demands a simultaneous treatment of electronic structure, interfacial solvation, ionic distributions, and potential‐dependent charge transfer, all of which operate across different length and time scales. Standard methods based on density functional theory, while powerful, must be extended or coupled with additional frameworks to account for the thermodynamic, electrostatic, and dynamic aspects of the electrochemical environment.

Over the years, theoretical approaches to electrocatalysis have evolved to address the complexity of the electrochemical interface. Initial treatments largely relied on thermochemical reaction models, evaluating reaction free energies under the assumption of concerted proton–electron transfers, most notably in the computational hydrogen electrode framework [5, 6, 7]. This approach enabled extensive screening of catalyst materials and provided mechanistic insights into key electrochemical reactions. However, its simplifications, such as neglect of activation barriers, treatment of electrode potential only a posteriori, and oversimplified or totally missing solvent representations, limit its accuracy and predictive power. More recent approaches attempt to incorporate solvation explicitly, model interfacial electric fields, or simulate potential‐dependent phenomena using electronically grand‐canonical formulations or advanced sampling techniques. The field has grown in sophistication, but also in diversity: no single method yet captures the full complexity of the electrified interface in a fully consistent and accurate way. Each model focuses on specific contributions such as adsorption energetics, electric field effects, solvent structure, or charge redistribution, and thus requires careful integration to construct physically meaningful simulations.

This review does not aim to catalog every available method. For comprehensive overviews of specific techniques and theoretical developments, the reader is referred to several excellent reviews available in the literature, such as [8, 9, 10, 11, 12, 13, 14, 15]. Instead, we focus on a set of guiding questions: how do theorists construct useful models of electrocatalytic systems? What approximations are typically made, and under what assumptions? Which features of the physical interface are routinely simplified and how might they be systematically reintroduced to improve predictive power? In exploring these questions, we seek not only to summarize the state of the art, but to clarify the logic behind it.

Our focus is on the theoretical foundations and practical implementations of electronic‐structure methods for modeling electrocatalytic systems. We discuss approaches to reaction thermodynamics, the treatment of electric bias, models of solvation, and various strategies for describing interfacial kinetics. In addition, we highlight recent developments such as the use of machine‐learning force fields and data‐driven descriptors tailored to electrochemical environments. By examining the assumptions and choices embedded in current methodologies, we aim to clarify their range of validity and point toward more robust and predictive frameworks for simulating the electrochemical interface.

To introduce the essential context for this discussion, we begin by briefly outlining the key physical concepts underlying electrochemical interfaces and atomistic modeling techniques. These elements provide the foundational framework for the methodological approaches examined in the sections that follow.

### 1.1 Electrochemical Interfaces

The electrochemical transformations we will discuss all take place at the interface between a solid electrode and a liquid electrolyte. This electrified region is a complex environment where electrons, ions, and solvent molecules interact across multiple spatial and temporal scales. From a modeling perspective, it presents a considerable challenge: a realistic description must account not only for chemical bonding and charge transfer at the atomic level, but also for electrostatic screening, solvation, and the coupling between electronic and ionic reservoirs under applied bias.

**FIGURE 1:**
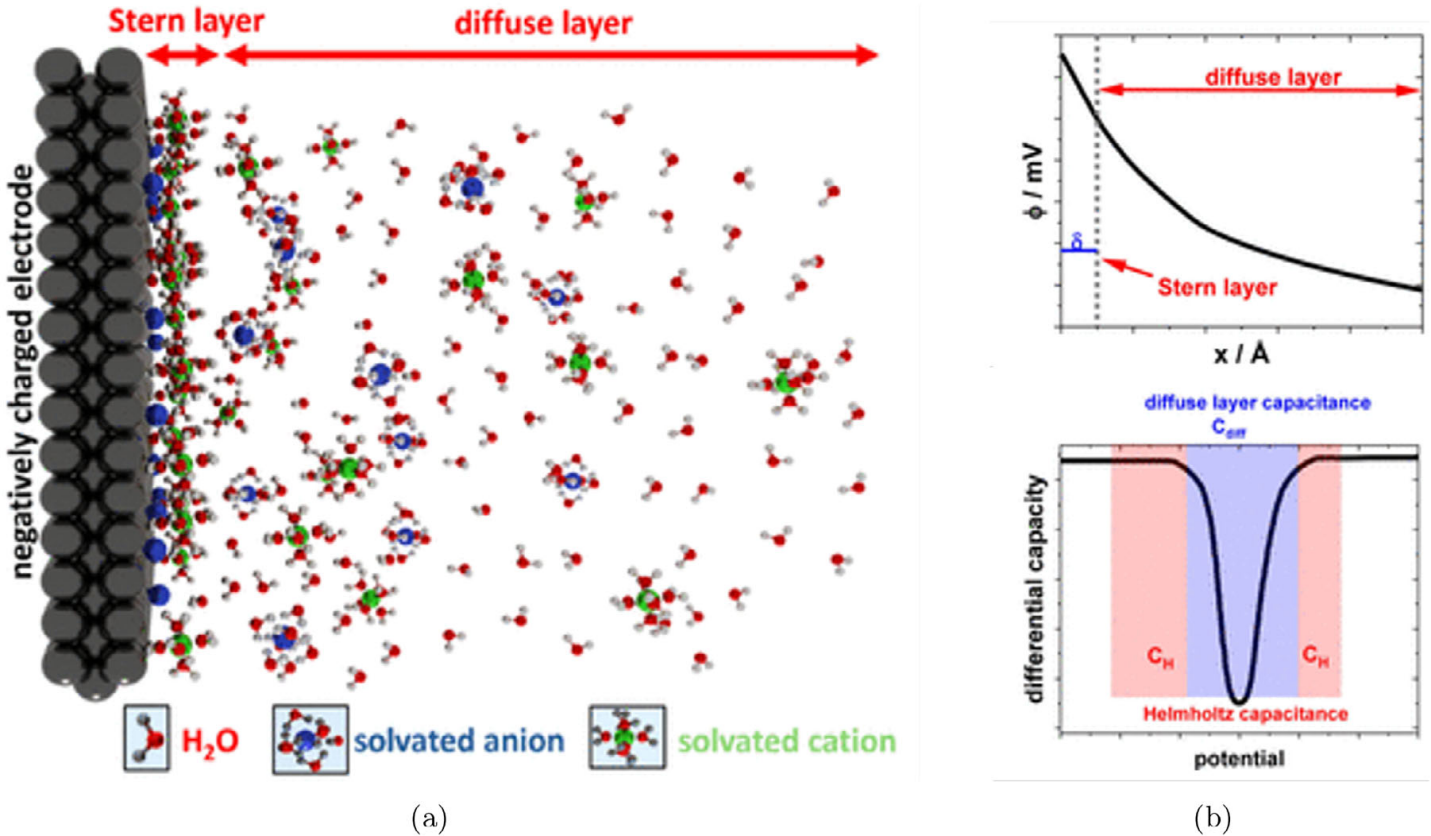
The electrochemical double layer. (a) Schematic representation of the solid/liquid interface and the EDL. (b) Upper panel: electrostatic potential profile across the EDL. Lower panel: differential capacitance of the EDL with diffuse layer and Helmholtz contributions. Reproduced under the terms of the CC‐BY 4.0 license [16]. Copyright 2024, The Authors, published by American Chemical Society.

At the heart of electrified solid/liquid interfaces lies the electrochemical double layer (EDL) [17, 18], shown schematically in Figure 1a. The EDL forms in response to surface charging of the electrode, which redistributes nearby ions and solvent molecules in the electrolyte to restore electroneutrality. This restructuring generates a spatially varying electrostatic potential, upper panel of Figure 1b, that governs the energetics of species at the interface. The resulting profile reflects a balance between the ordering effect of the electric field and the entropic tendency of thermal motion.

The EDL is typically divided into two main regions: a compact layer, also referred to as the Stern layer, and a diffuse layer that extends into the bulk electrolyte. The Stern layer itself is composed of two structurally distinct zones. The inner Helmholtz plane contains specifically adsorbed ions and partially desolvated molecules in direct contact with the electrode surface. These species interact chemically with the surface and define the innermost boundary of the EDL. The outer Helmholtz plane lies slightly farther from the surface and consists of solvated ions that are electrostatically attracted but not specifically adsorbed. Beyond the Stern layer lies the diffuse layer, where ions redistribute in response to the surface potential, and the electrostatic potential gradually relaxes toward its bulk value. The total charge in the EDL exactly compensates the excess charge on the electrode, preserving electroneutrality.

The electrode charge and interfacial structure of the EDL together determine the electrode potential, which sets the energy scale for electron transfer and influences the stability of charged and polar adsorbates at the interface. In experiments, this potential is measured relative to a reference electrode, such as the standard hydrogen electrode (SHE), and reported on a chosen scale [19]. In computational modeling, the electrode potential is linked to the electrochemical potential of electrons, which corresponds to the Fermi level of the electrode, appropriately referenced [20]. Closely related is the concept of the potential of zero charge (PZC), defined as the potential at which the net surface charge of the electrode vanishes. The PZC provides a natural reference for interpreting trends in surface charging, capacitance, and interfacial dipoles.

The ability of the interface to accommodate changes in charge in response to variations in potential is quantified by the (differential) double‐layer capacitance, lower panel of Figure 1b. This capacitance reflects both the electronic polarizability of the electrode and the dielectric response of the electrolyte and is sensitive to the structure and composition of the EDL. Metals, which have a high density of electronic states at the Fermi level, typically show large interfacial capacitance, whereas semiconductors often display more complex, potential‐dependent behavior due to band bending, surface states, or functionalization [20].

Beyond the EDL lies the diffusion layer, where concentration gradients arise due to finite ion transport and ongoing redox reactions. While electrically neutral, this layer plays a decisive role in determining the observed current–potential response, especially under dynamic or mass‐transport‐limited conditions. Its thickness depends on the timescale and hydrodynamic conditions of the experiment, and is typically much larger than that of the EDL [21].

Overall, the electrochemical interface is not a sharply defined boundary but a continuum of overlapping physical regimes: from quantum‐mechanical charge transfer at the electrode surface, through the structured solvent and ion layers of the Stern region, to long‐range ionic migration in the diffusion layer and the bulk electrolyte. The interplay of these components determines reaction pathways, energy barriers, and electrocatalytic activity. For computational studies aiming to simulate realistic interfacial processes, a physically grounded and consistent treatment of this structure is essential.

From an experimental perspective, substantial progress has been made toward the microscopic characterization of the EDL under operando and in situ conditions. Interface‐sensitive spectroscopic techniques, including surface‐enhanced infrared absorption spectroscopy (SEIRAS), surface‐enhanced Raman spectroscopy (SERS), and vibrational sum‐frequency generation (VSFG), provide direct access to the structure, orientation, and hydrogen‐bonding network of interfacial water as a function of electrode potential [22, 23, 24]. These measurements yield experimentally accessible observables, such as vibrational frequency shifts, intensity variations, polarization‐dependent responses, and Stark tuning rates, which can be directly compared with theoretical predictions of interfacial electric fields, water orientation distributions, and hydrogen‐bond strengths.

Complementary insight into the EDL is provided by scanning probe techniques operated under electrochemical control. Electrochemical atomic force microscopy (EC‐AFM) enables nanoscale mapping of force–distance relations and local dielectric properties across the interface, providing information on the thickness, compressibility, and layering of the Stern region [25, 26]. Such measurements offer direct points of comparison with density profiles and interfacial structuring obtained from theoretical simulations of electrified interfaces. Structural information at the atomic scale is further accessible through surface X‐ray scattering techniques [27, 28], such as surface X‐ray diffraction and crystal truncation rod measurements, which allow the characterization of electrode surface relaxation, adsorbate ordering, and interfacial layering under electrochemical control. The obtained spectra can then be compared to theoretical ones produced through atomistic simulations [29].

In addition, laser‐induced temperature‐jump methods, including laser‐induced current (LICT) and potential transient techniques, probe the collective reorganization of ions and solvent molecules at the interface. These approaches enable the determination of key double‐layer descriptors such as the PZC and the potential of maximum entropy [30, 31, 32]. These quantities can be related to computed work functions, surface charge densities, capacitances, and free‐energy profiles at electrified interfaces.

For a more comprehensive overview of experimental approaches to the characterization of the EDL, including optical spectroscopies, surface X‐ray scattering, scanning probe techniques, and electrochemical benchmarks, several recent reviews provide a broad and critical assessment of the capabilities and limitations of current methodologies [16, 33, 34, 35]. Together, these experimental developments define an increasingly stringent set of benchmarks against which theoretical and computational models of electrified interfaces can be validated and refined.

### 1.2 Density Functional Theory

Central to most first‐principles modeling in electrocatalysis is Density Functional Theory (DFT), which has become the standard quantum‐mechanical method for describing the ground‐state properties of materials and molecules at the atomic scale. While continuum models and empirical force fields play useful supporting roles, it is DFT that provides the basis for nearly all modern computational studies of electrocatalytic processes, particularly those focused on reaction energetics, adsorption phenomena, and surface chemistry. In practice, DFT provides the electronic structure data (total energies, charge densities, atomic forces) that are needed to construct and interpret atomic scale models of materials, molecules and reactions.

DFT is grounded in the Hohenberg–Kohn theorems [36], which establish that the ground‐state energy of an interacting many‐electron system is a unique functional E[ρ(r)] of the electron density ρ(r), and that the ground‐state energy can be obtained variationally by minimizing E[ρ(r)] with respect to ρ. This foundational result replaces the full many‐body wavefunction, which depends on 3N spatial coordinates for N electrons, with a scalar function of three variables, the electron density. The practical implementation of DFT is based on the Kohn–Sham formulation [37], which introduces an auxiliary system of non‐interacting electrons moving in an effective potential. The total energy is decomposed into a set of known contributions (kinetic, electrostatic, external potential) and a remaining unknown part: the exchange‐correlation (XC) energy, which captures all many‐body effects beyond the independent‐particle approximation.

In the absence of an exact expression for the XC energy functional, approximations are required. The most widely used classes of approximated XC functionals include the local density approximation (LDA), generalized gradient approximations (GGA) such as PBE [38] and RPBE [39], more advanced functionals like meta‐GGAs (e.g., SCAN [40], r^2^SCAN [41]), hybrid functionals like PBE0 [42] or HSE06 [43], and van der Waals–corrected schemes such as DFT‐D3 [44] or BEEF‐vdW [45]. Each of these approximations entails trade‐offs between accuracy, transferability, and computational cost. For electrocatalysis, GGA functionals remain dominant due to their efficiency and reasonable performance for adsorption energies and surface chemistry, though they may struggle to describe weak interactions, solvation, and certain charge‐transfer processes with sufficient accuracy. In such cases, meta‐GGA or hybrid functionals can be used, albeit at a larger computational cost.

DFT calculations are most commonly implemented using either plane‐wave basis sets, as in codes such as VASP [46], Quantum ESPRESSO [47, 48] and ABINIT [49], or localized atomic orbitals, as in SIESTA [50], Gaussian [51] and CRYSTAL [52]. Approaches using a dual basis set of atom‐centered Gaussian orbitals and plane waves are also possible, as implemented in CP2K [53]. Plane‐wave methods, typically combined with pseudopotentials or projector augmented‐wave techniques, as, for instance, in GPAW [54], are well suited for periodic systems and allow for easier convergence with respect to the basis set. Localized basis sets offer greater flexibility for open boundary conditions or reduced‐dimensionality models, and are often used in molecular or cluster calculations. In both cases, periodic boundary conditions are standard, even for surface and interface simulations. This simplifies the treatment of extended systems and enables the use of efficient reciprocal‐space algorithms, but also introduces artifacts in systems involving net charges or electric fields.

Standard DFT calculations describe the ground state of a fixed number of electrons at zero temperature, providing total ground‐state energies that can then be transformed to free energies by accounting for entropic and enthalpic terms. Vibrational contributions can be included through the harmonic approximation, while finite‐temperature effects can be treated using statistical mechanics, atomistic thermodynamics or molecular dynamics [55].

DFT has proven remarkably successful in reproducing and predicting a wide range of experimental observables, from binding energies of adsorbates and surface reconstructions to activation barriers and reaction pathways. Numerous comprehensive reviews and books cover the theoretical foundations, numerical implementation, and practical considerations of DFT in much greater detail than we do here. The reader is referred to refs. [56, 57, 58, 59, 60, 61, 62] for broad introductions and technical overviews.

In the context of electrocatalysis, DFT provides the essential input for describing surface reactivity at the atomic scale. Most commonly, it is used to compute adsorption energies and reaction free energies for intermediates on metal or semiconductor surfaces. These outputs form the basis of thermodynamic models of electrochemical reactions, discussed in Section 2. To capture the influence of solvation and the electrochemical environment, DFT can be combined with different types of solvent models, reviewed in Section 3. The treatment of electrode potential, a defining feature of electrochemical systems, requires additional methodological developments examined in Section 4. For modeling reaction kinetics, DFT supplies the potential energy surface over which transition‐state searches are performed and activation energies are calculated, as presented in Section 5. Finally, DFT data provide the primary training set for machine‐learning models and interatomic force fields. They are also used to compute reaction descriptors for high‐throughput screening. Both applications are central to the emerging field of data‐driven electrocatalysis, as discussed in Section 6.

In this review, DFT is thus treated not as a self‐contained method, but as the foundational engine that underlies, and often limits, theoretical approaches to modeling the electrochemical interface. Each of the following sections explores how DFT is embedded within broader modeling frameworks, what assumptions are made in doing so, and what kinds of predictions can or cannot be trusted.

## 2 Thermochemical Methods

As mentioned in the introduction, a first approach to computational electrocatalysis relies on thermochemical methods, notably the computational hydrogen electrode. These methods treat electrochemical reactions as a sequence of proton‐coupled electron transfers (PCETs), evaluated through total Gibbs free energies of intermediates and gas‐phase or solvated species. Thermochemical methods are simple, effective, conceptually clear, and make smart use of equilibrium thermodynamics to bypass the explicit treatment of electrode potentials and solvated ions. However, they come with important limitations that the theoretical modeler must recognize, manage, and, when necessary, address through more advanced approaches.

### 2.1 The Computational Hydrogen Electrode

One of the main challenges in first‐principles modeling of electrochemical reactions is the treatment of the electrochemical potentials of electrons under applied bias and solvated protons. These quantities are difficult to define and compute explicitly within standard electronic‐structure methods. The computational hydrogen electrode (CHE), introduced by Nørskov and co‐workers [5, 6, 7], circumvents this problem by replacing the direct treatment of electrons and solvated protons with a well‐defined thermodynamic reference based on the hydrogen redox couple.

The central idea behind the CHE is to exploit the thermodynamic equilibrium at the SHE: at standard conditions (298 K, 1 bar, pH = 0), the half‐cell reaction

(1) $$H_{\left(\mathrm{aq}\right)}^{+}+e^{-}⇌\frac{1}{2}H_{2}\left(g\right)$$

is in equilibrium. This implies that the electrochemical potential of a proton–electron couple at the SHE is

(2) $${\tilde{\mu }}_{H^{+}}^{\circ }+{\tilde{\mu }}_{e}\left(U=0\right)=\frac{1}{2}\mu_{H_{2}}^{\circ }$$

here, ${\tilde{\mu }}_{H^{+}}^{\circ }$ and $\mu_{H_{2}}^{\circ }$ are the electrochemical and chemical potentials of a solvated proton and hydrogen molecule in the gas phase at standard conditions, respectively. By definition, ${\tilde{\mu }}_{e}\left(U=0\right)$ is the electrochemical potential of an electron at 0 V with respect to the SHE. At an arbitrary potential U versus SHE, ${\tilde{\mu }}_{e}$ is shifted by −eU, ${\tilde{\mu }}_{e}\left(U\right)={\tilde{\mu }}_{e}\left(U=0\right)-eU$, yielding

(3) $${\tilde{\mu }}_{H^{+}}^{\circ }+{\tilde{\mu }}_{e}\left(U\right)=\frac{1}{2}\mu_{H_{2}}^{\circ }-eU$$

The effect of pH is introduced via the Nernst equation, which adjusts the electrochemical potential of a proton in the electrolyte as

(4) $${\tilde{\mu }}_{H^{+}}\left(\text{pH}\right)={\tilde{\mu }}_{H^{+}}^{\circ }+k_{B}T\ln\left[H^{+}\right]={\tilde{\mu }}_{H^{+}}^{\circ }-k_{B}T\ln10\times \text{pH}$$

The electrochemical potential of the proton–electron couple at arbitrary pH and potential U versus SHE is then

(5) $${\tilde{\mu }}_{H^{+}}+{\tilde{\mu }}_{e}\left(U\right)=\frac{1}{2}\mu_{H_{2}}^{\circ }-eU-k_{B}T\ln10\times \text{pH}$$

By grouping together the U and pH dependencies, one can define the potential $U_{\mathrm{RHE}}$ relative to the reversible hydrogen electrode (RHE) as

(6) $$\begin{array}{cc} U_{\text{RHE}} & =U+\frac{k_{B}T}{e}\ln10\times \text{pH} \end{array}$$

(7) $$\begin{array}{cc} & ≃U+0.059\;V\times \text{pH} \end{array}$$

where T=298 K, so that

(8) $${\tilde{\mu }}_{H^{+}}+{\tilde{\mu }}_{e}\left(U\right)=\frac{1}{2}\mu_{H_{2}}^{\circ }-eU_{\mathrm{RHE}}$$

Equation (5) or Equation (8) allow for the straightforward calculation of the Gibbs free energy change associated with a PCET at any applied potential versus SHE or RHE, respectively. A representative example is the Volmer step in the hydrogen evolution reaction (HER),

(9)

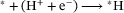

where * denotes a surface adsorption site and *H a chemisorbed hydrogen atom. The corresponding Gibbs free energy change at an electrode potential U versus SHE is given by

(10)

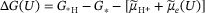

here, $G_{*}$ and 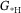 are the Gibbs free energies of the clean surface and the hydrogen‐covered surface, respectively. Notice that the electrode potential U enters in ΔG only in the dependence ${\tilde{\mu }}_{e}\left(U\right)$ and not in the Gibbs free energies $G_{*}$ and 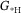. These quantities can be evaluated using standard, neutral‐cell DFT calculations complemented with atomistic thermodynamics [63, 64, 65]. For instance,

(11)

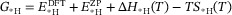

where $E^{\mathrm{DFT}}$ is the total electronic energy from DFT, $E^{\mathrm{ZP}}$ is the zero‐point energy, and ΔH(T) and S(T) are the enthalpy and entropy contributions. For adsorbates on surfaces, these quantities are commonly derived from vibrational normal modes within the harmonic approximation [65], treating all degrees of freedom as vibrational.

Under the CHE assumption, the sum of the proton and electron electrochemical potentials in Equation (10) can be replaced via Equation (5) or Equation (8), giving

(12)

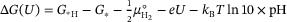



(13)

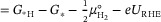


$\mu_{H_{2}}^{\circ }$ is readily obtained from the DFT total energy of an $H_{2}$ molecule, supplemented with standard thermochemical corrections available in databases such as the NIST Chemistry WebBook [66] or the JANAF Thermochemical Tables [67].

This example illustrates how the free energy change of any reaction step involving a PCET can be directly referenced to gas‐phase hydrogen and adjusted by the applied potential U versus SHE and pH or, equivalently, by the applied potential $U_{\mathrm{RHE}}$ versus RHE.

**FIGURE 2:**
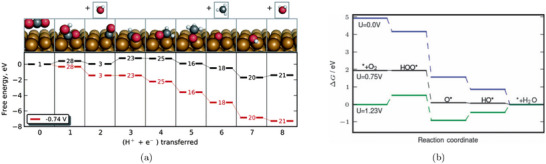
Gibbs free energy profiles of electrochemical reduction reactions computed with the CHE. (a) ${\mathrm{CO}}_{2}\mathrm{RR}$ to ${\mathrm{CH}}_{4}$ on the Cu(211) surface with geometries and Gibbs free energies of the various reaction steps. The Gibbs free energy profile is computed at zero applied potential (black lines) and at U=−0.74 V versus RHE (red lines). Reproduced with permission [68]. Copyright 2010, The Royal Society of Chemistry. (b) ORR on Pt(111) at three different applied potentials. Gibbs free energy paths are computed at U=0 versus RHE (blue line), at the limiting (onset) potential U=0.75 V versus RHE (black line) and at the thermodynamic equilibrium potential U=1.23 V versus RHE (green line). Reproduced with permission [7]. Copyright 2008, PCCP Owner Societies.

Once the free energy change of each step of a given reaction is computed following this approach, one can construct a reaction free energy diagram. This enables the identification of thermodynamic bottlenecks along the reaction path. The key quantity extracted from such a diagram is the reaction onset (limiting) potential $U_{\text{onset}}$, defined as the most positive potential at which all PCET steps along the reaction pathway become thermodynamically downhill

(14) $$U_{\text{onset}}=\max_{i}\left\{\Delta G_{i}\left(U=0\right)/e\right\}$$

where $\Delta G_{i}\left(U=0\right)$ is the free energy change of the i‐th step at zero applied potential. In Figure 2a, we show an example of such free energy diagrams for the electrochemical reduction of ${\mathrm{CO}}_{2}$ (${\mathrm{CO}}_{2}\mathrm{RR}$) to methane on the Cu(211) surface [68]. The thermodynamic limiting step is the hydrogenation of adsorbed *CO to *CHO, step 2→3 in Figure 2a, which requires the largest free energy input. This step is brought to equilibrium by applying a bias of $U_{\mathrm{RHE}}=-0.74$ V (red lines), making all steps downhill in free energy.

A similar analysis for the oxygen reduction reaction (ORR) on Pt(111) [7] is shown in Figure 2b. The study identifies two nearly degenerate potential‐determining steps: the formation of adsorbed hydroperoxyl (*OOH) from gas‐phase $O_{2}$, and the desorption of hydroxyl (*OH) to form water.

Both steps become thermodynamically downhill at a potential close to 0.80 V versus RHE. This value is lower than the experimentally observed ORR onset on Pt(111), which is typically reported in the range 0.9–1.0 V versus RHE [69, 70, 71]. This discrepancy illustrates a common limitation of CHE‐based predictions, which often exhibit an offset relative to experimentally observed onsets under realistic electrochemical conditions. Such deviations highlight the need for approaches that go beyond the traditional CHE framework.

The success of the CHE approach and its widespread application lie in its conceptual clarity and computational efficiency, as it sidesteps the explicit treatment of electrons and solvated protons. Nevertheless, the CHE involves critical approximations that must be carefully considered. Most notably, the CHE in its basic formulation neglects the dynamic nature of the solvent, the structure of the EDL, the influence of the interfacial electric field, and reaction kinetics.

The plain CHE framework evaluates adsorbed species in vacuum, neglecting both solvent effects and the presence of the EDL. While accurately capturing the EDL requires more advanced approaches (see for instance Section 6.4), solvent interactions with adsorbates can be approximately accounted for through different methods, as discussed in Section 3.

Reaction intermediates extending into the EDL experience electrostatic forces that depend on the applied potential. Intermediates with significant dipole moments, like *COOH or *OOH, or those that protrude into the electrolyte are particularly sensitive to local electric fields, which can affect adsorption energies by several tenths of an eV [72].

In its standard formulation, the CHE assumes that proton and electron transfer occur simultaneously, i.e., as a concerted step. This assumption breaks down in reaction mechanisms involving decoupled charge transfer events, such as sequential electron‐then‐proton or proton‐then‐electron steps, where intermediate charge‐separated states may be stabilized. The explicit treatment of electrode polarization and charged species is therefore essential. The most commonly used methods to incorporate these effects are discussed in Section 4.

Finally, the CHE is a purely thermodynamic approach and does not consider reaction kinetics. Energy barriers for PCET steps are assumed to be negligible and are therefore entirely excluded from the analysis [5]. As a consequence, the CHE framework provides an incomplete description of the reaction mechanism and may predict preferred pathways that deviate significantly from those observed experimentally, particularly when activation barriers are rate‐limiting. Strategies for incorporating kinetics and estimating activation energies are discussed in Section 5.

### 2.2 Gas‐Phase Corrections

Discrepancies between theoretical predictions and experimental results can arise already at the level of CHE‐based reaction free energy calculations. A well‐known source of error lies in the systematic inaccuracies of DFT‐computed molecular energies, particularly for small molecules such as $O_{2}$, CO, ${\mathrm{CO}}_{2}$, and various nitrogen‐ and oxygen‐containing species, often involved in technologically‐relevant electrochemical processes. GGA functionals, such as PBE, RPBE, and BEEF‐vdW, tend to overbind or underbind these molecules, leading to deviations of 0.2–0.6 eV in computed standard formation energies [73, 74]. These errors propagate through reaction thermodynamics and can significantly skew predictions of free‐energy diagrams, as well as equilibrium and onset potentials.

Several correction schemes have been developed to address this issue. A common strategy is empirical fitting of DFT formation energies against experimental thermodynamic data [68]. These errors are then attributed to specific molecules or functional groups (e.g., –${\mathrm{CH}}_{x}$, –C═O, –COOH) and used as additive corrections. Group‐additivity approaches decompose the total DFT error into transferable motif‐specific components, enabling systematic corrections across chemical families. For example, Granda‐Marulanda et al. [73] developed a correction scheme based on a dataset of 27 molecules from the carbon cycle. They identified systematic DFT functional errors of approximately +0.24 eV for CO and −0.19 eV for ${\mathrm{CO}}_{2}$, along with group‐wise deviations for common functional groups: corrections of around +0.03 eV for –${\mathrm{CH}}_{x}$ and −0.10 eV for –C═O moieties.

More advanced schemes make use of bond‐matrix representations or functional group ensembles to isolate sources of error [75]. Corrections can also be inferred from key formation reactions (e.g., $H_{2}O$ and ${\mathrm{NH}}_{3}$ synthesis), thus obtaining the energies of problematic species through known thermodynamics. Regardless of methodology, these corrections have been shown to reduce mean absolute errors in reaction energies by over an order of magnitude [73], and improve the accuracy of derived descriptors such as limiting potentials, activity volcanoes, and scaling relations.

## 3 Inclusion of Solvation Effects

DFT calculations performed in vacuum completely neglect the complex environment in which electrocatalytic reactions occur. One of the most immediate missing effects is solvation. Solvent interactions are essential to electrochemical systems, for example through the stabilization of charged or polar intermediates via hydrogen bonding with water. To obtain physically meaningful reaction energetics, solvation effects must therefore be properly accounted for.

In the following, we review several strategies for incorporating solvation effects, varying in both complexity and accuracy. In general, systematic comparisons among these different strategies on application‐relevant systems are not common. For instance, ref. [76] compares vacuum calculations, ad hoc solvation corrections, implicit solvation models, and explicit micro‐solvation approaches, showing progressively improved agreement with experimental onset potentials as more realistic solvation effects are included.

### 3.1 Ad Hoc Corrections

Solvation at the solid–liquid interface can be most simply incorporated by applying ad hoc corrections to the Gibbs free energies of reaction intermediates computed within the CHE. These corrections are typically obtained from differences in adsorption energies computed with and without a small number of explicit solvent molecules, from experimentally or computationally determined enthalpies of formation, or from averaged hydrogen‐bond energies [5, 68, 77, 78, 79, 80, 81]. Reported values range from –0.1 to –0.6 eV for various adsorbates involved in the electroreduction of $O_{2}$, ${\mathrm{CO}}_{2}$, and NO. For example, hydroxyl adsorbates (*OH) and indirectly bound hydroxyl species (*ROH) were stabilized by approximately 0.5 eV [5, 82] and 0.25 eV [77], respectively, on Pt(111) in the presence of water. Similarly, on Cu(111), *OH in a half‐dissociated water layer was stabilized by 0.58 eV, while *COH and *CO were stabilized by 0.38 and 0.07 eV, respectively [78]. However, *ROH, where R is a hydrocarbon chain, exhibited a stabilization of 0.38 eV on Cu(111), differing from that of Pt(111).

In general, ad hoc solvation corrections show a strong dependence on the electrode surface and the nature of the adsorbate [83, 84, 85], making their estimation nontrivial and potentially leading to significant errors in predicted reaction energetics.

### 3.2 Implicit Solvation Models

**FIGURE 3:**
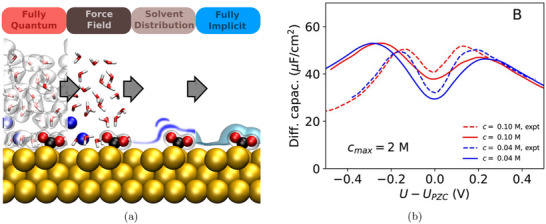
Implicit solvation. (a) Hierarchy of solvation models, ranging from fully explicit quantum‐mechanical descriptions (left) to fully implicit continuum models (right). Intermediate levels include classical force fields and statistical or density‐based solvent representations. Reproduced under the terms of the CC‐BY 4.0 license [87]. Copyright 2021, The Authors, published by American Chemical Society (b) Differential capacitance of Ag(100) in a ${\mathrm{KPF}}_{6}$ electrolyte solution. The simulated capacitance curves (solid lines) are obtained by solving the mPB equation with $c_{\max}=2$ M, see Equation (18), and the SSCS boundary. Dashed lines are experimental measurements. Reproduced with permission [89]. Copyright 2019, AIP Publishing.

A more systematic and physically grounded alternative to model the surrounding liquid environment is implicit solvation, which coarse‐grains the electrolyte into a continuous polarizable medium (see Figure 3a) [86, 87, 88].

Implicit solvation models have become essential in computational electrocatalysis, as they allow the simulation of electrochemical environments without representing individual solvent molecules or ions. By replacing the explicit solvent with a dielectric continuum and, when needed, incorporating electrolyte effects, these models provide a tractable yet physical approximation. Their efficiency and flexibility make them particularly well‐suited for simulating extended systems such as charged metal surfaces under applied potential.

Implicit solvation models extend the DFT energy functional E[ρ(r)] of the system to a free energy functional [88]

(15) $$G\left[\rho \left(r\right)\right]=E\left[\rho \left(r\right)\right]+G^{\mathrm{elec}}\left[\rho \left(r\right)\right]+G^{\text{non}\text{-elec}}\left[\rho \left(r\right)\right]$$

by adding an electrostatic energy functional $G^{\mathrm{elec}}\left[\rho \left(r\right)\right]$ that accounts for the electrostatic interactions between the solute and the solvent, incorporating the characteristics of the solvating medium, such as its dielectric permittivity and ion concentrations, and a non‐electrostatic term $G^{\text{non}\text{-elec}}\left[\rho \left(r\right)\right]$ that includes all other interactions between the solute system and the solvent. The functional G[ρ(r)] is then variationally minimized to obtain the energy and charge density. To build G[ρ(r)], implicit solvation must define the interface between the quantum‐mechanical solute and the continuum solvent. One of the earliest and most widely used approaches is the Polarizable Continuum Model (PCM), which separates solute and solvent using a boundary formed by the union of atom‐centered hard spheres, typically based on van der Waals radii [90, 91]. This construction imposes a sharp dielectric discontinuity at the solute–solvent interface and does not respond to changes in the electronic structure of the solute. While the PCM is effective for molecular systems, particularly for estimating solvation energies of small neutral or ionic species, it is less appropriate for extended or metallic systems, where surface polarization and charge redistribution play a central role and require a more adaptive interface.

A more flexible alternative is the class of isodensity‐based models. These define the dielectric function ε(r) as a smooth function of the local electronic density ρ(r). The Self‐Consistent Continuum Solvation (SCCS) model [92, 93] uses a switching function s(ρ) to interpolate between vacuum and bulk permittivity $\varepsilon_{\text{bulk}}$,

(16) $$\varepsilon \left[\rho \left(r\right)\right]=1+\left(\varepsilon_{\text{bulk}}-1\right)s\left(\rho \left(r\right)\right)$$

This allows the boundary to adapt self‐consistently to field‐induced charge redistributions, crucial for modeling electrode charging.

Another commonly employed framework for the solvent‐solute boundary is the Soft Sphere Continuum Solvation (SSCS) model [94], that offers a different strategy by building the solute cavity from overlapping Gaussian‐smeared atomic spheres. SSCS is less responsive to electronic changes than SCCS but performs well for molecular or hybrid systems, and is particularly suited to FFT‐based solvers.

A known limitation of many continuum solvation models is their tendency to allow the solvent to penetrate regions that are physically inaccessible to real solvent molecules, such as narrow pores, surface recesses, or sterically crowded areas. This problem has been effectively addressed by solvent‐aware models, which introduce nonlocal corrections that suppress the dielectric response in confined regions [95]. These corrections improve robustness, especially in rough or porous geometries, and yield a more realistic representation of the solute–solvent boundary, helping stabilize geometry optimizations and molecular dynamics at complex interfaces. These implicit solvation models are available in several widely used electronic structure codes, including VASP via the recently developed VASPsol++ plugin [96], Quantum ESPRESSO through the ENVIRON module [93], and JDFTx [97].

For realistic modeling of electrochemical systems, the dielectric response of the solvent must be coupled to an electrolyte description. This is typically achieved by extending the Poisson equation to a Poisson‐Boltzmann (PB) equation, which includes the contribution of mobile ions through Boltzmann‐distributed concentrations

(17) $$c_{i}\left(r\right)=\gamma \left(r\right)c_{i}^{0}\exp\left(-\frac{z_{i}e\phi \left(r\right)}{k_{B}T}\right)$$

where $c_{i}^{0}$ is the bulk concentration of ion species i, $z_{i}$ its valence, and ϕ(r) the electrostatic potential. The exclusion function γ(r) ensures that ions are confined to the solvent‐accessible region and do not penetrate the solute interior. When the electrostatic potential is small relative to the thermal energy, the Boltzmann factor in Equation (17) can be expanded to first order, giving a linear dependence on ϕ(r) and leading to the linearized Poisson–Boltzmann (LPB) equation.

While the PB or LPB equations capture the mean‐field screening effect of ions, they assume point‐like ions and become inaccurate at high surface potentials or ion concentrations. In particular, they predict unphysically large ion densities near highly charged surfaces, violating the finite volume available for packing real ions. To address this, the modified Poisson–Boltzmann (mPB) equation introduces steric constraints that account for the excluded volume of the ions. These corrections impose a saturation limit on the total ionic concentration, preventing divergence of the charge density in the vicinity of the electrode. A common form of the size‐modified model adjusts the ion concentration as [98, 99]

(18) $$c_{i}\left(r\right)=\frac{\gamma \left(r\right)c_{i}^{0}\exp\left(-\frac{z_{i}e\phi \left(r\right)}{k_{B}T}\right)}{1+\sum_{j}\frac{c_{j}^{0}}{c_{\max}}\left[1-\exp\left(-\frac{z_{j}e\phi \left(r\right)}{k_{B}T}\right)\right]}$$

where $c_{\max}$ sets the maximum local concentration allowed by steric constraints. This form ensures that ionic crowding is properly limited and yields more realistic ionic profiles, especially near charged surfaces. In the limit $c_{\max}\to +\infty$ or for vanishing ionic radius, the mPB reduces to the standard PB equation.

These ionic models enable the computation of surface charge, interfacial fields, and differential capacitance. The latter varies with the potential depending on the structure of the EDL. The more advanced mPB models allow for asymmetric or camel‐shaped capacitance curves, capturing effects of ion concentration, asymmetry, and permittivity. These features can be compared directly with experimental capacitance data and are critical for interpreting potential‐dependent reaction energetics. Figure 3b compares differential capacitance profiles computed by solving the mPB equation, with the SSCS solute–solvent boundary, against experimental measurements [89]. The electrochemical system consists of an Ag(100) surface in a ${\mathrm{KPF}}_{6}$ electrolyte. The maximum ion concentration in Equation (18) is set to $c_{\max}=2$ M, and results are shown for two bulk concentrations, $c^{0}=0.10$ M and $c^{0}=0.04$ M. The computed profiles capture both the position of the PZC and the overall shape of the experimental curves.

A crucial practical consequence of including an electrolyte model in the simulations is the enforcement of overall charge neutrality in the cell. The ions in the implicit electrolyte redistribute in response to electrostatic fields, effectively screening any net charge present on the electrode or introduced through explicit ionic species. As a result, the total electrostatic potential remains well‐defined, and spurious interactions between periodic images are avoided. This feature enables simulations of polarized electrodes, where the metal slab carries a net charge, as well as systems containing explicit charged species, such as hydronium, hydroxide, or ionic intermediates involved in reaction steps. Moreover, most implementations automatically reference the Fermi level of the quantum‐mechanical system to the bulk electrolyte, allowing for a direct estimation of the electrode potential (see Section 4.2). The electrolyte thus plays a dual role: it captures the physical screening behavior of the electrochemical environment and ensures consistency in simulations of charged interfaces.

Despite their efficiency, implicit solvation models are intrinsically limited in their ability to describe short‐range, directional solvent–adsorbate interactions. In particular, hydrogen bonding between water molecules, adsorbates, and the electrode surface is not explicitly captured, leading to a systematic underestimation of the stabilization of polar intermediates. This limitation has been demonstrated, for instance, in benchmark studies on Pt(111), where continuum solvation models were shown to significantly underestimate the solvation energies of *OH and related species compared to explicit solvent descriptions, with consequences for predicted onset potentials and reaction energetics [100, 101]. While implicit approaches generally improve upon vacuum calculations by accounting for long‐range electrostatic screening, they do not reproduce the site‐specific stabilization arising from hydrogen‐bond networks at the solid–liquid interface, motivating the need for explicit solvation approaches.

### 3.3 Explicit Solvation Models

By replacing the discrete solvent with a continuum dielectric medium, implicit solvation captures only the macroscopic polarization response, while neglecting specific molecular interactions. As a result, important local effects, such as hydrogen bonding between solvent molecules and adsorbed intermediates (e.g., *OH or *OOH in ORR), may be missed or only roughly approximated [102, 103]. Explicit solvation approaches can, in principle, overcome this limitation. By modeling solvent molecules directly at the quantum level, they can capture both the collective dielectric response and local interactions with reactive species. However, such methods are typically highly computationally demanding, requiring large simulation cells and extensive configurational sampling.

A first minimal approach to capture hydrogen bonding and solvation energetics is micro‐solvation [76]. This method determines the stabilizing contribution of each water molecule in the solvation shell by comparing its interaction with the adsorbate versus its interaction with the surrounding water. The solvation shell of the adsorbate is constructed by sequentially adding individual water molecules and relaxing the system after each step. Only those molecules that interact preferentially with the adsorbate are retained, resulting in a minimal, explicit solvent environment with clear physical relevance. This approach aligns well with previous work showing that the first solvation shell often suffices to estimate solvation energies. Despite its neglect of solvent dynamics, this strategy reproduces experimental trends in reaction intermediates and onset potentials with good accuracy [104].

**FIGURE 4:**
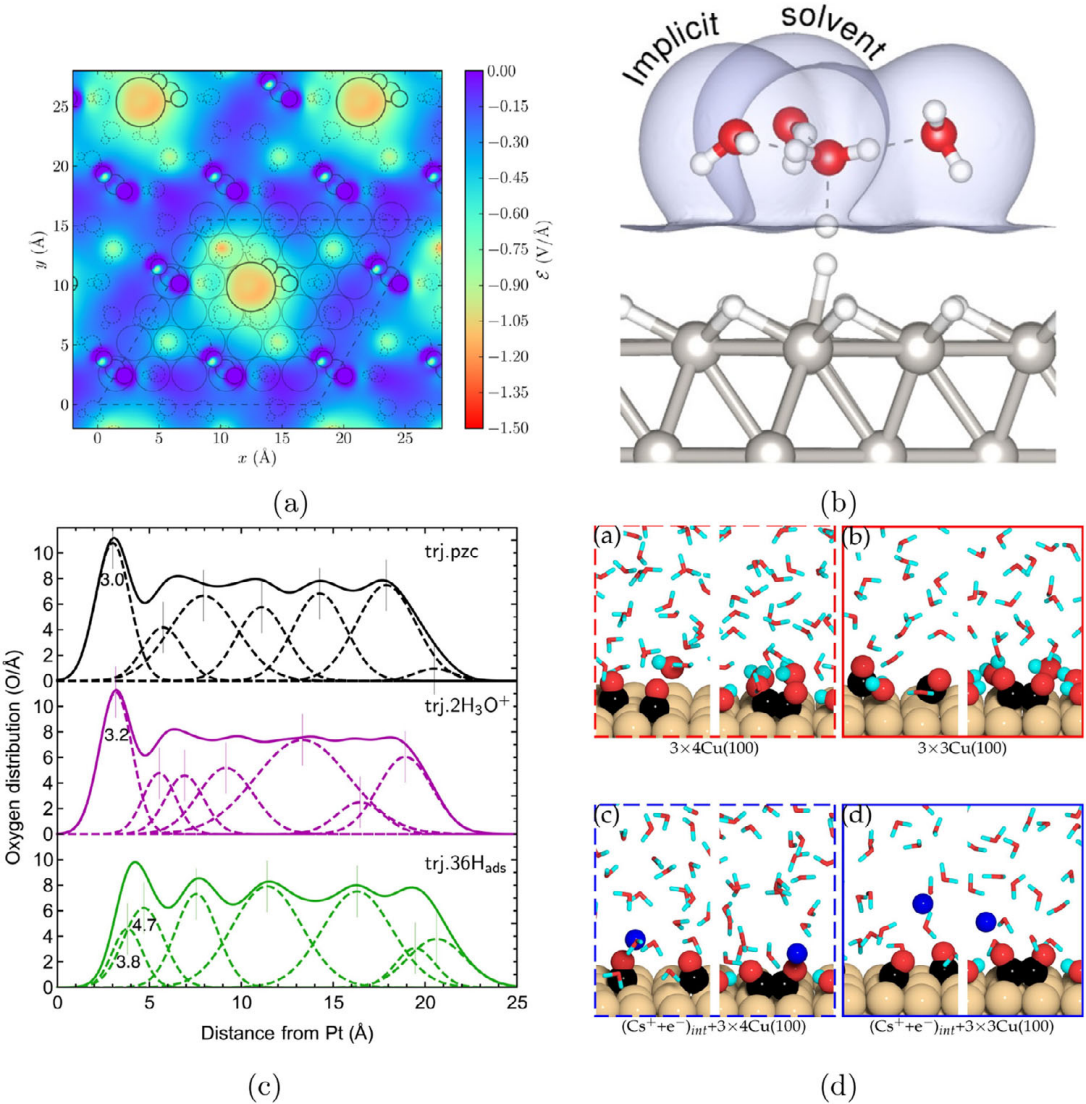
Explicit solvation. (a) Electric field distribution evaluated on a slice parallel to an Ag(111) electrode, near an adsorbed *${\mathrm{CO}}_{2}$ in a supercell with a $K^{+}$ (large circles) cation solvated by explicit water molecules. Reproduced with permission [80]. Copyright 2016, American Chemical Society. (b) Hybrid explicit‐implicit solvation of a hydronium ion on a Pt(111) surface. The isosurface represents the solute‐solvent boundary. Reproduced with permission [113]. Copyright 2019, American Chemical Society. (c) Distribution of oxygen atoms in the water film on Pt(111) via AIMD simulations with slightly different conditions: pure water (upper panel), water with two hydronium ions (middle panel), and pure water on Pt fully covered by hydrogen atoms (lower panel). Reproduced with permission [114]. Copyright 2018, AIP Publishing. (d) Snapshots of 2 *CO and *OCCO adsorbed on the Cu(100) surface in different supercell sizes with and without Cs^+^ cations. Reproduced with permission [115]. Copyright 2021, Elsevier Inc.

To better capture solvation effects, larger static solvent structures are often employed. One approach identifies global minimum solvent configurations using constrained minima hopping [105], which combines constrained molecular dynamics (MD) with local relaxation. This method has been applied to several electrocatalytic processes, including proton–electron transfer and CO dimerization steps relevant to ${\mathrm{CO}}_{2}$ reduction [106, 107]. Alternatively, local minimum solvent structures, constructed with a small number of explicit water molecules, offer a balance between accuracy and efficiency. This approach has been shown to yield solvation energies within 0.15 eV of fully sampled models when 5–10 water molecules are included [108]. Calculations involving a few water molecules have been used to evaluate thermodynamics and kinetics of the ${\mathrm{CO}}_{2}$ reduction reaction (${\mathrm{CO}}_{2}\mathrm{RR}$) on Cu(100) and Cu(111) [109], as well as electric field effects in CO_2_RR to CO on Ag(111) in the presence of a solvated cation layer, as shown in Figure 4a. The heatmap shows the magnitude of the electrostatic field originated by the introduction of a K^+^ ion (large circle) in the simulation supercell [80]. The cation is solvated by an explicit water layer and its presence stabilizes the adsorption of *${\mathrm{CO}}_{2}$ (smaller circles) on the catalyst surface.

More elaborate setups, including explicit bilayer ice structures with hydronium ions [110, 111] or multi‐layer water configurations to account for surface–water and water–water interactions [112], have also been employed in studies of CO_2_RR to multi‐carbon products.

Static explicit solvent structures can be combined with implicit solvation to capture both local adsorbate/electrolyte interactions and the macroscopic response of the liquid phase [113, 116, 117]. In this hybrid approach, a small number of explicit water molecules, typically forming a first solvation layer, are placed directly near the electrode surface to account for microscopic interactions such as hydrogen bonding. Above this explicit layer, an implicit solvent model takes over, describing the macroscopic dielectric and ionic response of the surrounding electrolyte, as schematically shown in Figure 4b. An explicit water cluster, solvating a hydronium ion, is placed on a Pt(111) surface with hydrogen coverage, while the space above the cluster is filled with implicit solvent [113]. Because modern implicit models are solvent‐aware (see Section 3.2), they naturally exclude interstitial regions in between explicit solvent molecules, avoiding any unphysical overlap. This combination yields a physically consistent and computationally tractable framework for simulating electrochemical interfaces under realistic conditions, while preserving key atomistic features of solvent–adsorbate interactions.

To move beyond static solvent structures and include the dynamical behavior of the liquid phase, MD simulations can be combined with explicit solvation. MD provides a natural way to sample the configurational space of the solvent, capturing thermal fluctuations, hydrogen‐bond rearrangements, and transient interactions with adsorbates. This dynamical treatment is particularly important for understanding solvation shells, ion distributions, and time‐dependent interfacial phenomena. While classical MD is valuable for sampling solvent structure and achieving equilibration [118, 119, 120], its inability to describe even basic water chemisorption [121, 122], or more generally, to account for bond‐breaking and bond‐forming events, fundamentally limits its applicability to reactive electrochemical systems.

Processes such as PCET, adsorption of intermediates, and chemical transformations at the electrode surface involve strong coupling between the electrolyte, solvent, and catalyst surface, and therefore require a fully quantum‐mechanical treatment [121, 122]. In such cases, ab initio molecular dynamics (AIMD) provides a natural framework, as it enables an accurate, dynamic description of both the electronic structure and thermal motion of the system. AIMD allows for the simultaneous sampling of solvent configurations and electronic structure, providing a rigorous framework to capture solvent–adsorbate interactions at finite temperature.

Figure 4c illustrates how AIMD, after classical pre‐equilibration, captures the structuring of interfacial water in Pt(111) under various conditions: pure water near the point of zero charge, water with hydronium ions and water on hydrogen‐covered Pt. The resulting oxygen density profiles reveal strong layering effects and shifts in peak positions that reflect changes in the electrochemical environment [114]. AIMD has also been applied extensively to electrochemical systems. For example, studies of CO reduction on Cu(100) at pH 7, in the presence of explicit water, successfully revealed reaction pathways in agreement with experimental data [123].

More complex systems, such as CO–CO coupling on Cu(100) with co‐adsorbed Cs^+^ ions, have also been investigated (Figure 4d), employing supercells with 30 H_2_O molecules to simulate the solid–liquid interface [115]. The presence of Cs^+^ was shown to slightly lower the activation barrier for CO dimerization (see Section 5.3). AIMD simulations have also been used to evaluate the role of different electrolytes, including aqueous and phosphate buffer solutions across a range of pH values, on the stabilization of CO reduction intermediates [124]. While AIMD, possibly enhanced through advanced sampling techniques (see Section 5.3), provides rich atomistic insight, its high computational cost limits both the time scales and the system sizes that can be practically explored. Hybrid quantum/classical approaches (Section 3.3.1) and machine‐learning‐based force fields (Section 6.4) have emerged as promising alternatives, offering improved accuracy and transferability while enabling simulations over longer time scales and larger system sizes.

From a practical standpoint, AIMD applied to electrocatalytic systems involves numerous technical subtleties in the simulation setup. These typically reflect compromises between accuracy and tractability, yet their implications are often under‐discussed.

A key issue arises when simulating solvents that contain light atoms, particularly hydrogen. Due to its low mass, hydrogen exhibits high vibrational frequencies, which require a reduced timestep, typically below 0.25 fs, to maintain integration stability. However, such short timesteps significantly limit the accessible simulation timescale. A widely adopted workaround is to substitute hydrogen with its heavier isotope, deuterium, allowing a timestep of 0.5 fs without compromising numerical stability [55]. While this substitution has negligible effects on equilibrium and thermodynamic properties, it can distort dynamical quantities such as vibrational spectra and diffusion coefficients.

Another important consideration is the choice of thermostat. Standard thermostats such as Nosé–Hoover may perform poorly in heterogeneous systems like catalyst/solvent interfaces, where significant temperature gradients can emerge across different regions of the simulation cell [125]. More advanced algorithms, including Nosé–Hoover chains [126] and stochastic velocity rescaling (i.e., Bussi–Donadio–Parrinello) [127], often provide a more reliable temperature control and should be used when available.

The predictive accuracy of AIMD is further limited by the intrinsic shortcomings of the underlying DFT XC functional. One of the most persistent challenges is the accurate treatment of the interplay between covalent bonding, hydrogen bonding, and dispersion forces, that collectively determine the structure of liquid water and the solvation environment around adsorbates [128]. The commonly used PBE functional, for instance, is known to over‐structure water [129]. Various corrections have been proposed to address this issue, including empirical dispersion schemes (e.g., PBE+D3, rPBE‐D3) [44], van der Waals functionals such as BEEF‐vdW [45], and more recent meta‐GGA approaches like SCAN [40]. Still, overstructuring remains a general problem across functionals. A widespread workaround is to increase the simulation temperature above ambient conditions (e.g., 400 K for PBE, 330 K for SCAN) to compensate for this artifact. While this practice can reproduce some experimental features of water, it should be viewed as an empirical fix that does not address the deeper limitations of the DFT functionals, nor the neglect of nuclear quantum effects (NQEs) [130, 131].

NQEs refer to the breakdown of the classical point‐particle approximation for atomic nuclei, particularly for light elements such as hydrogen. In such cases, a quantum mechanical treatment of nuclear motion becomes necessary. These effects can substantially influence properties such as zero‐point energy, tunneling, and isotope‐dependent behavior. Neglecting these factors in the simulation of electrocatalytic interfaces has an impact on the strength of the hydrogen bonds in water, resulting in overstructuring the H_2_O network, altering the solvation shells and overestimating the proton transfer barriers [131, 132]. Strategies to incorporate NQEs at affordable computational cost are discussed in Section 6.4.

#### 3.3.1 Quantum Mechanics/Molecular Mechanics (QM/MM)

As highlighted in the previous section, AIMD simulations have proven valuable for gaining insight into the complex environment of electrocatalytic interfaces. However, they are computationally demanding, particularly when the goal is to obtain a quantitative estimate of solvation energy, the effect of applied voltage and charge transfer at the electrode–electrolyte interface [133, 134, 135]. AIMD simulations are typically limited to a timescale of only a few picoseconds, whereas sampling over nanoseconds is often necessary to achieve proper equilibration and convergence of statistical averaging [136, 137].

Atomic scale simulations of electrochemical reactions at liquid/solid interfaces are particularly challenging. Accurate treatment of bond breaking and formation requires quantum mechanical (QM) electronic structure methods, such as DFT, where the computational effort increases rapidly with system size. At the same time, proper statistical sampling of the liquid phase demands a large simulation cell and long timescale trajectories, pushing the computational cost beyond practical limits for AIMD. To overcome these challenges, the hybrid quantum mechanics/molecular mechanics (QM/MM) strategy is a promising approach. Such a hybrid scheme was originally developed by Warshel and Levitt over fifty years ago to model enzymatic reactions [138]. In a QM/MM simulation the system is partitioned into two regions. The chemically active region, where bond rearrangements and electronic effects take place, is treated using an electronic structure method. The remaining part of the system is described using a potential energy function depending only on atomic coordinates. This partitioning scheme dramatically reduces computational effort for large systems while retaining the accuracy needed to describe reactive events at the catalytic site. QM/MM can be a powerful tool for simulating electrocatalytic reactions in a realistic way.

The total energy of the system is split into the energy of each subsystem (QM and MM) and the explicit interaction between them (QM/MM)

(19) $$E^{\mathrm{tot}}=E^{\mathrm{QM}}+E^{\text{QM}\text{/MM}}+E^{\mathrm{MM}}$$

There are several levels of the QM/MM approach that can be categorized by how the QM and MM subsystems are coupled together and interact. The three primary schemes are: mechanical embedding (ME), electrostatic embedding (EE), and polarizable embedding (PE).

In ME‐QM/MM calculations, the interaction between the QM and MM regions is described with a potential energy function with no explicit account of the effect that the long range electrostatic field from the MM region has on the electronic structure of the QM region. Consequently, the QM charge density does not respond to the surrounding electrostatic environment. While computationally efficient, ME is poorly suited for modeling electrochemical interfaces as it does not capture the electronic polarization effects.

In EE‐QM/MM calculations, the MM environment is represented as an array of point charges that are incorporated into the Hamiltonian of the QM subsystem. The resulting electrostatic energy is given by

(20) $$E^{\text{QM}\text{/MM}}=-\sum_{j}^{N_{\mathrm{MM}}}q_{j}\int \frac{\rho (r)}{|r-R_{j}|}dr+\sum_{j}^{N_{\mathrm{MM}}}\sum_{i}^{N_{\mathrm{QM}}}\frac{q_{j}Z_{i}}{|R_{i}-R_{j}|}+E_{\mathrm{NE}}$$

where $q_{j}$ is the point charge of atom j at position $R_{j}$, ρ(r) is the electronic density of the QM subsystem, $Z_{i}$ is the charge of nucleus i at position $R_{i}$, and $E_{\mathrm{NE}}$ is the interaction energy from the remaining non‐electrostatic interaction terms [139, 140]. In this way, the electronic density of the QM subsystem can respond to an electrostatic field generated by the MM region, and thereby the influence of the electrolyte solution on interfacial reactions can be captured to some extent. The EE‐QM/MM method has been used to estimate the free energy of water at the Pt(111)/water interface [141] and to study the HER on ${\mathrm{MoS}}_{2}$ electrodes [142]. These studies demonstrate the practicality of the EE‐QM/MM approach for long‐timescale dynamics, with simulations spanning up to 6 nanoseconds and the solvent represented by over 1000 water molecules. Such a timescale and system size remain computationally inaccessible to AIMD simulations. However, because the point charges of the atoms in the MM region are fixed and do not respond to changes in the QM charge distribution, EE‐QM/MM does not describe mutual polarization and can underestimate critical effects such as the formation of the electrical double layer, which extends into the electrolyte solution and represents the response to the electric field at the electrode.

The PE‐QM/MM approach overcomes the limitations of the ME and EE embeddings by explicitly accounting for mutual polarization between the QM and MM subsystems. Polarizabilities are assigned to MM molecules (or auxiliary expansion points), allowing the MM environment to dynamically respond to the electronic density of the QM region. The QM charge distribution induces dipoles and higher multipoles in the molecules of the MM subsystem, and these in turn contribute to the QM Hamiltonian, resulting in a self‐consistent polarization field. Consequently, the total energy of the system is expressed as a functional of both the QM electronic density and the induced polarization of the MM environment. For simplicity, the discussion here is limited to induced dipoles and the energy of the system can then be written as

(21) $$E^{\mathrm{tot}}\left[\rho \left(r\right),\Delta \mu_{\alpha }^{i}\right]=E^{\mathrm{QM}}\left[\rho \left(r\right)\right]+E^{\text{QM}\text{/MM}}\left[\rho \left(r\right),\Delta \mu_{\alpha }^{i}\right]+E^{\mathrm{MM}}\left[\Delta \mu_{\alpha }^{i}\right]$$

where the coupling between the QM electron density and the MM environment can be expressed as an interaction energy functional,

(22) $$E_{\text{el}\text{.+ind.}}^{\mathrm{QM}/\mathrm{MM}}=\int \rho \left(r\right)V^{\mathrm{MM}}\left(r\right)dr$$

with ρ(r) being the charge density of the QM subsystem and $V^{\mathrm{MM}}\left(r\right)$ the electrostatic potential generated by the MM environment, including both permanent and induced multipoles. For a dipolar expansion, the MM potential at position r can be written as a sum over MM sites i, which, in Einstein notation, reads

(23) $$V^{\mathrm{MM}}\left(r\right)=\sum_{i}T_{\alpha }^{ri}\left(\mu_{\alpha }^{i}+\Delta \mu_{\alpha }^{i}\right)$$

where $T_{\alpha }^{ri}$ is the dipole interaction tensor connecting the charge density at r to site i, $\mu_{\alpha }^{i}$ the permanent dipole moment, and $\Delta \mu_{\alpha }^{i}$ is the induced dipole moment at that site. This interaction can equivalently be expressed as a sum over contributions produced by the QM charge density at the MM sites

(24) $$E_{\text{el}\text{.+ind.}}^{\mathrm{QM}/\mathrm{MM}}=\sum_{i}\left(\mu_{\alpha }^{i}+\Delta \mu_{\alpha }^{i}\right)V_{\alpha }^{i\mathrm{QM}}$$

where the potential field (negative of the electric field) at site i due to the QM charge density is given by

(25) $$V_{\alpha }^{i\mathrm{QM}}=\int \rho \left(r\right)T_{\alpha }^{ri}dr=\int \rho \left(r\right)\left(-\frac{1}{r^{3}}r_{\alpha }\right)dr$$

This, in turn, induces the dipole moment via

(26) $$\Delta \mu_{\alpha }^{i}=-\alpha_{\alpha \beta }^{i}\left(V_{\beta }^{i\mathrm{MM}}+V_{\beta }^{i\mathrm{QM}}\right)$$

where $\alpha_{\alpha \beta }^{i}$ is the dipole–dipole polarizability, and $V_{\alpha }^{i\mathrm{MM}}$ is the potential field at site i due to all other MM sites. This self‐consistent treatment captures the dynamic coupling between charged species and interfacial fields, making PE‐QM/MM particularly well‐suited for simulating electrochemical systems where dielectric screening, solvation, and field effects play important roles. Sophisticated PE‐QM/MM implementations including electrical dipoles have been developed in the context of electronic excitations of solvated molecules [143, 144, 145, 146, 147, 148, 149, 150, 151]. Higher order expansions up to the hexadecapole, which have been found to be adequate for typical intermolecular distances in water [152], have also been presented [153, 154].

While the PE‐QM/MM approach provides a more rigorous treatment of interfacial polarization than mechanical or electrostatic embedding, it has so far not been applied to electrocatalysis. PE‐QM/MM frameworks have only recently been extended to solid/liquid interfaces and their implementation in standard simulation packages is still maturing, partly due to the lack of transferability of polarizable force fields. A step in the direction of electrocatalysis is the calculation of the Raman frequencies of some surface‐bound intermediates in ${\mathrm{CO}}_{2}\mathrm{RR}$ [155]. There, the Cu(100)/water interface was modeled by a system consisting of a 4×4 Cu surface slab covered by a 2 nm‐thick explicit water layer (72 water molecules), enabling a description of interfacial polarization. By accounting for the polarization of the solvent, the simulation was able to accurately describe shifts in the vibrational peaks.

Careful partitioning of the QM and MM subsystems is essential to accurately describe the interaction between the electrode and the electrolyte solution. While the bulk solution can be described using MM potential functions, solvent molecules and ions closest to the electrode surface must be treated at the QM level to capture processes such as charge transfer, hybridization of electronic states at the solid–liquid interface, solvent–adsorbate interactions, and critical elementary steps such as hydrogen adsorption and proton transfer. However, a key challenge arises because the QM and MM solvent molecules and ions tend to diffuse across the QM/MM boundary. To address this, various partitioning schemes have been developed [156, 157, 158, 159, 160, 161, 162, 163, 164, 165, 166, 167, 168, 169], broadly falling into two categories: adaptive and constraining approaches. Adaptive boundary schemes gradually switch between the QM and MM description of the diffusing species in a broad interface region. This is computationally demanding because it requires several simultaneous QM and MM evaluations to maintain continuity of energy and atomic forces [166, 167, 168]. In contrast, constraining schemes assign the QM and MM particles to their respective part of the system, using either a fixed or flexible location of the boundary, and thereby prevent exchange between the two regions [157, 158, 159, 169]. This approach avoids the computational cost of adaptive schemes and is therefore more practical for large and long timescale electrocatalytic simulations. For example, in the SAFIRES method, the interface is determined by the position of the outermost QM molecule and an abrupt scattering event takes place if an MM molecule attempts to enter the QM region [169]. This method has been shown to give accurate statistical sampling, while the dynamical trajectories of molecules are clearly not correct. To avoid artifacts due to the perturbed trajectories, the QM/MM interface is chosen to lie far enough from the active region.

Another critical challenge in PE‐QM/MM simulations is to ensure that the potential energy function used to describe molecules in the MM region yields electrostatic properties that are consistent with the electronic structure method used for the QM region. For example, the MM potential function should reproduce the electrostatic properties of the density functional chosen in the DFT calculation of the QM region, even if it does not represent the best estimate compared to experimental observations. Inconsistencies across the interface lead to artifacts at the QM/MM boundary. Potential energy functions with high‐level description of the electrostatics, and thereby transferable to different environments, have been developed for water and acetonitrile [153, 154, 170, 171, 172]. These potential functions are typically parameterized by fitting results of quantum mechanical calculations (such as energy and atomic forces, multipole moments, and polarizabilities) of the solvent molecules at a specified level of theory.

## 4 Applied Electric Bias

Thermochemical methods (see Section 2) owe their simplicity to avoiding explicit treatment of solvated protons and electrode polarization. However, this simplification is also a major limitation, as realistic simulations require the applied bias to be included explicitly. Below, we discuss two main approaches to account for electrode polarization, which can ultimately be shown to be equivalent in the thermodynamic limit.

### 4.1 Constant‐Charge Approaches

Electrochemical processes occur at constant electrode potential, yet periodic DFT simulations are normally performed at fixed electron number, i.e., at constant charge [173]. This fundamental discrepancy introduces challenges when modeling charge‐transfer reactions at electrode–electrolyte interfaces. In a fixed‐charge setup, transferring an electron between the electrode and the electrolyte changes the system work function, effectively altering the electrode potential during the reaction. This artificial variation of the electrode potential leads to unphysical changes in the computed reaction energies and activation barriers, especially in small unit cells.

This issue is particularly severe when solid/liquid interfaces are simulated in periodic supercells, where a single charge‐transfer event is replicated across the entire surface due to the boundary conditions. As a result, the electrochemical reaction is modeled as occurring simultaneously at every unit cell, producing a large collective change in the surface dipole and potential. In some cases, the resulting bias change during the reaction can exceed several volts, distorting both the thermodynamics and kinetics.

To reconcile constant‐charge simulations and experimentally relevant constant‐potential conditions, the cell‐extrapolation scheme provides a systematic method to correct for the artificial potential change that arises during charge‐transfer reactions in periodic DFT [173, 174]. This method is based on a physically motivated capacitor model of the electrochemical interface and aims to recover reaction energetics at fixed electrode potential by compensating for finite‐size artifacts.

To charge the electrode, species such as alkali metals (K, Na, Cs) or H_3_O· radicals are typically introduced into the simulation cell. These readily ionize, transferring an electron to the electrode and remaining as positively charged species in the EDL. As a result, the electrode acquires a surface charge density θ and a corresponding electrostatic potential U. Increasing the number of added species raises the overall electrode charge. The Gibbs free energy of a reaction intermediate can be interpreted as the energy stored in a capacitor, where the charge on the plates corresponds to the ions in the EDL and the counter‐charge on the electrode. As such, the energy per unit area can be approximated as a quadratic function of the electrode potential, $g=C{\left(U-U_{\mathrm{pzc}}\right)}^{2}/2=\theta^{2}/2C$, where $U_{\mathrm{pzc}}$ is the PZC (used as reference), C is the interfacial capacitance per unit area, θ is the surface charge density on the electrode and U the corresponding electrode potential.

**FIGURE 5:**
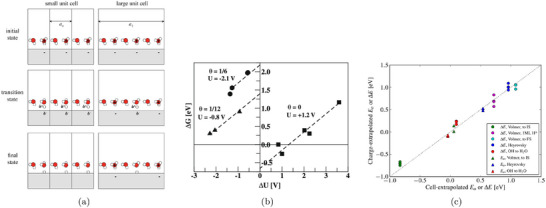
Constant‐charge approaches to explicit electric bias. (a) Schematic representation of the surface charge variations with increasing supercell size. Reproduced with permission [175]. Copyright 2016, American Chemical Society. (b) Reaction Gibbs free energy ΔG for the Heyrovsky step in HER as a function of potential change ΔU, computed for varying supercell sizes and surface charge densities θ. Reproduced with permission [173]. Copyright 2008, Elsevier B.V. (c) Parity plot comparing reaction energies, ΔE, or activation energies, $E_{a}$, of different reaction steps (see legend) obtained with the cell‐extrapolation (x‐axis) and the charge‐extrapolation (y‐axis) schemes. Reproduced with permission [175]. Copyright 2015, American Chemical Society.

When modeling a charge‐transfer step with standard canonical DFT, the surface charge density θ changes by Δθ, resulting in a corresponding shift ΔU in the electrode potential between the reactant, transition, and product states. This variation in surface charge along the reaction coordinate is schematically illustrated in Figure 5a [175].

Considering the capacitor model, when the total charge is n and one unit charge is transferred from one plate to the other, the energy changes as

(27) $$\begin{array}{cc} \Delta G & =A\left[g(A,n-1)-g(A,n)\right]=Ae^{2}\left[{\left(\theta -1/A\right)}^{2}-\theta^{2}\right]/2C \end{array}$$

(28) $$\begin{array}{cc} & =-eU-1/2e\Delta U \end{array}$$

where A is the surface area of the cell, U=−eθ/C, and ΔU is the finite‐size‐induced potential shift, which scales as 1/A. Within this capacitor picture, the dominant contribution to the potential variation along a reaction step is expected to be electrostatic, and thus approximately proportional to ΔU [173]. As shown in Figure 5b, plotting the computed energies versus ΔU yields a linear trend. Each point corresponds to a calculation performed in a supercell of different size at fixed surface charge density θ. Different values of θ correspond to different applied potentials U and give distinct trends (circles, triangles and squares in the picture). Extrapolating each trend to the ΔU=0 limit provides an estimate of the reaction energetics in the thermodynamic (infinite‐cell) limit at the corresponding electrode potential U.

In practice, and with reference to Figure 5b, the extrapolation procedure can be summarized as follows:

(i) For a chosen initial surface charge density θ (here reported as the coverage of ions in the simulation cell, e.g. θ=1/6, θ=1/12, or θ=0 in Figure 5b), construct a sequence of increasing lateral supercells (e.g., 3×2, 6×2, 6×4) describing the same initial and final states of the elementary step.
(ii) For a given θ, all supercells are computed at the same electrode potential U, which is obtained from the work function $\Phi_{i}$ of the initial state. The electrode potential is related to the work function via
(29) $$U=(\Phi -\Phi_{\mathrm{SHE}})/e$$
  where $\Phi_{\mathrm{SHE}}$ denotes the work function of the standard hydrogen electrode. In Figure 5b, for example, the family of supercells with θ=1/12 corresponds to an electrode potential of U=−2.1 V.
(iii) For each supercell, compute the work function $\Phi_{f}$ of the final state and define the potential change along the reaction step as
(30) $$\Delta U=(\Phi_{f}-\Phi_{i})/e$$
(iv) Compute the corresponding reaction free energy (or activation barrier) ΔG for each supercell at fixed θ.
(v) For each θ, plot the computed values of ΔG as a function of ΔU. Data corresponding to different surface charge densities naturally group into distinct families, as illustrated by the different symbols (circles, triangles, and squares) in Figure 5b.
(vi) Fit each family of points to a linear relation,
(31) ΔG(ΔU;θ)≈a(θ)ΔU+b(θ)
  as shown in Figure 5b. The constant‐potential estimate at the electrode potential associated with θ is then obtained from the intercept,
(32) ΔG(ΔU=0;θ)=b(θ)
  For instance, in Figure 5b, the system at U=−0.8 V (triangles), yields a constant‐potential reaction free energy of ΔG∼1.5 eV, obtained from the intersection of the fitting line and the vertical line at ΔU=0.

In summary, Figure 5b can be interpreted as a graphical recipe: for each surface charge density θ, the intercept at ΔU=0 yields the reaction energetics at fixed electrode potential, while the slope quantifies the magnitude of finite‐size effects inherent to constant‐charge simulations.

Performing simulations in increasingly large supercells to suppress finite‐size effects quickly becomes computationally prohibitive. To mitigate this, simplified models further exploiting the electrostatics of a parallel‐plate capacitor have been proposed [175, 176]. These approaches aim to reduce the number of required constant‐charge DFT calculations while still recovering accurate energetics through charge‐extrapolation.

The central assumption is that the total energy change along a reaction step can be separated into chemical and electrostatic contributions. The latter can be evaluated using a capacitor model of the interface. Under this assumption, the capacitive energy difference between two reaction states 1 and 2 is given by [175]

(33) $$\begin{array}{c} E_{\mathrm{cap},2}\left(U_{2}\right)-E_{\mathrm{cap},1}\left(U_{1}\right)=e\left(q_{2}-q_{1}\right)\left[-\left(U_{1}-U_{\mathrm{pzc}}\right)-\left(U_{2}-U_{1}\right)/2\right] \end{array}$$

where $q_{2}-q_{1}$ is the charge transferred between the two states, $U_{\mathrm{pzc}}$ is the PZC, $U_{1}$ and $U_{2}$ are the electrode potentials computed in states 1 and 2, respectively. The first term, proportional to $U_{1}$, corresponds to the capacitive energy change at constant potential $U_{1}$, i.e., $E_{\mathrm{cap},2}\left(U_{1}\right)-E_{\mathrm{cap},1}\left(U_{1}\right)$. The second term, proportional to the potential difference $U_{2}-U_{1}$, encodes finite‐size effects. The total energy change between states 1 and 2 at constant potential $U_{1}$, including both chemical and electrostatic contributions, can then be written as

(34) $$E_{2}\left(U_{1}\right)-E_{1}\left(U_{1}\right)=E_{2}\left(U_{2}\right)-E_{1}\left(U_{1}\right)+\frac{1}{2}e\left(q_{2}-q_{1}\right)\left(U_{2}-U_{1}\right)$$

This expression enables the estimation of constant‐potential energy differences from a single pair of DFT calculations for states 1 and 2, each performed at constant charge and thus at different electrode potentials $U_{1}$ and $U_{2}$. The final term in Equation (34) provides a correction for the spurious capacitive energy associated with the potential shift in finite‐size cells.

This charge‐extrapolation scheme is particularly straightforward to implement. Simulations of the two reaction states yield total energies and Fermi levels, which can be directly converted into electrode potentials U. The charge transferred between the two states, $q_{2}-q_{1}$, can be readily estimated using charge partitioning methods such as Bader analysis.

As shown in the parity plot in Figure 5c, results from the charge‐extrapolation method are consistent with those obtained from the cell‐extrapolation approach, while significantly reducing the computational cost [175].

### 4.2 Constant‐Potential Approaches

A fundamental limitation of conventional DFT‐based modeling of electrochemical systems is its use of the canonical ensemble, in which the number of electrons is fixed. This constraint prevents direct simulation of processes at constant electrode potential, which is the experimentally relevant condition for electrocatalysis. Electronically grand‐canonical density functional theory (GC‐DFT) addresses this problem by treating the electron number as a fluctuating variable and instead fixing the electrochemical potential of electrons, effectively modeling the system in the grand‐canonical ensemble where the electrochemical potential is externally controlled.

In GC‐DFT, the thermodynamic potential of interest is the grand potential

(35) $$\Omega \left({\tilde{\mu }}_{e}\right)=E-{\tilde{\mu }}_{e}N_{e}$$

where E is the total internal energy, obtained from DFT calculations, $N_{e}$ is the net number of electrons (relative to a neutral system), and ${\tilde{\mu }}_{e}$ is the electron electrochemical potential, directly related to the applied electrode potential. Once properly referenced, ${\tilde{\mu }}_{e}$ can be translated to any conventional electrode scale, such as the SHE, making the applied bias U explicit. For instance, one obtains

(36) $${\tilde{\mu }}_{e}=-e\left(U+\Phi_{\mathrm{SHE}}\right)$$

where $\Phi_{\mathrm{SHE}}≃4.44$ V is the absolute potential of the SHE [177].

The objective of the GC‐DFT simulation is to minimize Ω with respect to both the electron density and the electron number, while keeping ${\tilde{\mu }}_{e}$ constant. This can be achieved through iterative schemes that vary $N_{e}$ during SCF convergence [178, 179], or through outer‐loop algorithms that adjust $N_{e}$ based on feedback from the Fermi level or total potential, until a predefined target potential is reached. Recent work has demonstrated stable implementations of such fully converged constant‐potential methods using Newton's method and polynomial fitting to estimate the required derivatives for electron number updates [179].

All methods for adjusting $N_{e}$ depend on determining the electronic electrochemical potential ${\tilde{\mu }}_{e}$ and, via Equation (36), the corresponding electrode potential U. They also require a mechanism to counterbalance the modified electrode charge to maintain overall charge neutrality in the simulation cell.

**FIGURE 6:**
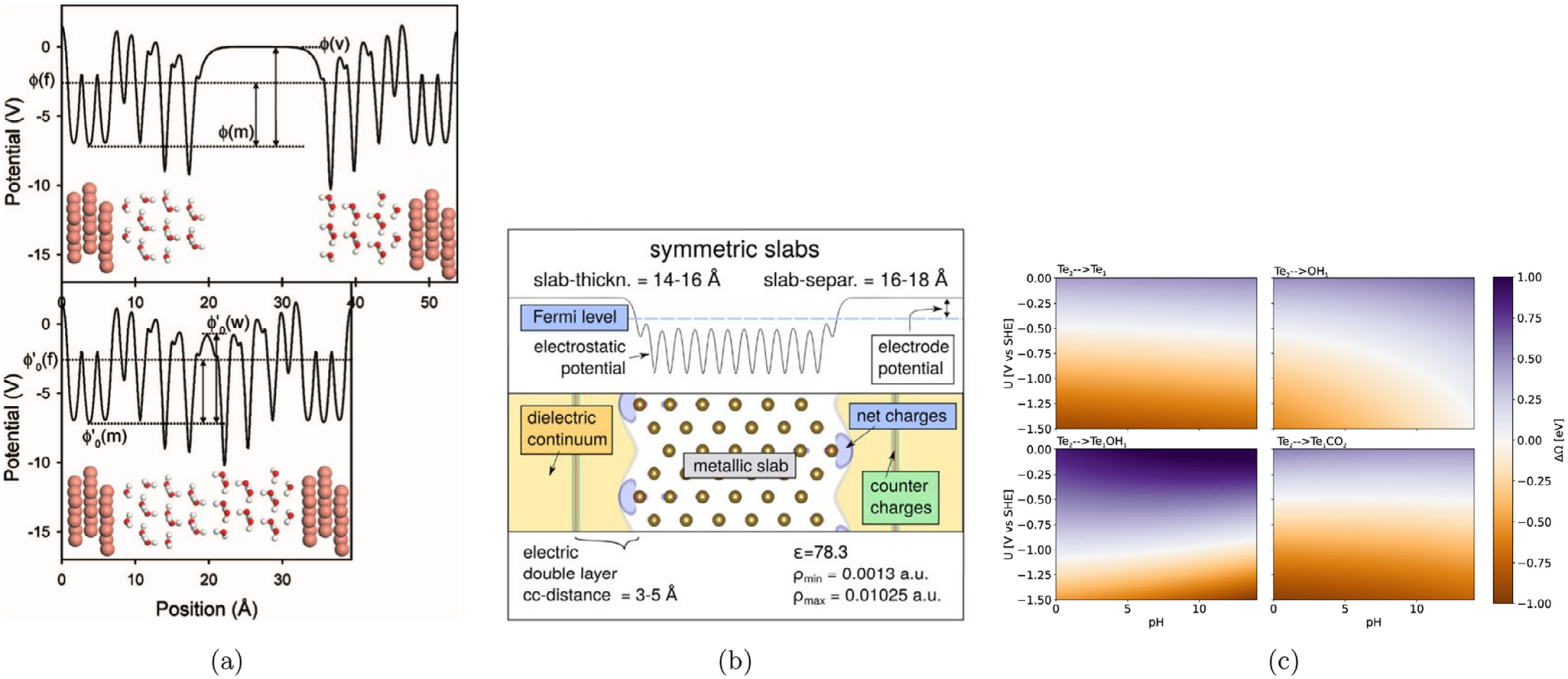
Constant‐potential approaches to explicit electric bias. (a) Double‐reference method. Upper panel, calculation with vacuum gap and determination of the potential levels ϕ(v) in the vacuum and ϕ(m) in the electrode. Lower panel, gapless calculation with determination of the bulk electrolyte potential ϕ(w). Reproduced with permission [180]. Copyright 2006, The American Physical Society. (b) Schematic representation of a typical computational setup with a metal slab immersed in an implicit solvent environment. Potential and Fermi energy alignments are also shown. Reproduced with permission [182]. Copyright 2019, AIP Publishing. (c) Heatmaps of the grand potential difference ΔΩ for various ${\mathrm{MoTe}}_{2}$ edge terminations relative to the ${\mathrm{Te}}_{2}$ termination, plotted as a function of potential versus SHE and pH. Reproduced with permission [185]. Copyright 2023, American Chemical Society.

A first method to control electrode potential in DFT simulations employs a homogeneous background charge to preserve charge neutrality when the number of electrons in the slab is varied [180, 181]. This so‐called double‐reference method introduces two reference levels for the electrode work function, enabling the determination of ${\tilde{\mu }}_{e}$ and, correspondingly, U on the absolute scale. A symmetric simulation cell is used, composed of a metal slab in contact with liquid water, and the electrostatic potential profile ϕ(z) is evaluated along the direction normal to the interface. A scheme of this setup is reported in Figure 6a. In a first step, a vacuum region is introduced by cleaving a portion of the bulk electrolyte. The electrostatic potential in this vacuum region, ϕ(v), is used as the vacuum level reference. The potential within the metal slab, ϕ(m), is then referenced to ϕ(v), upper panel of Figure 6a. In a second step, a calculation is performed without the vacuum gap, and the electrostatic potential in the bulk electrolyte, ϕ(w), is obtained. This value is referenced to the vacuum level through the previously determined ϕ(m), under the assumption that the potential in the electrode remains unchanged between the two setups, lower panel of Figure 6a. In this way, also the electrostatic potential in the electrolyte can be referenced to the vacuum level and placed on the absolute scale. When the number of electrons $N_{e}$ is varied, an electric field develops across the interface, which is screened by the electrolyte. The electrostatic potential in the bulk solvent, ϕ(w), remains constant, and can be used as a second reference level to extract the electrode work function in the charged state. Although the homogeneous background charge is unphysical, it has been shown that the screening properties of the electrolyte yield a realistic electrostatic potential profile in the inner double layer, consistent with that obtained using explicit counterions [180].

The double‐reference method is straightforward to implement and compatible with the standard treatment of background charge available in most DFT codes. It naturally includes explicit solvation (see Section 3.3), enabling a realistic description of interfacial structure and reactivity under bias. While it requires two separate calculations to establish the reference levels, making it somewhat more computationally demanding, it avoids the need for complex counterion placement and remains a robust and widely applicable approach.

A more recent implementation of a constant‐potential approach combines GC‐DFT with an implicit electrolyte model to ensure charge neutrality (see Section 3.2). In this approach, the electrolyte is represented as a polarizable dielectric continuum with a counter‐ion distribution that screens excess charge on the electrode [178, 182].

In most practical realizations, such as in VASPsol [183, 184], VASPsol++ [96], and ENVIRON [93], the Fermi level computed within the simulation cell is referenced to the electrostatic potential in the bulk solvent. Accounting for the potential offset between the bulk solvent and the vacuum level, the computed Fermi level gives ${\tilde{\mu }}_{e}$ referenced to the vacuum level. A standard computational setup including implicit solvation is shown in Figure 6b. Thanks to the ongoing development of DFT codes and compatible implicit solvation plugins, its large‐scale adoption is now becoming feasible. GC‐DFT enables accurate investigation of reaction thermodynamics, kinetics, and catalyst morphology under operating conditions. For example, GC‐DFT simulations enabled the construction of Pourbaix diagrams to assess the stability of different edge terminations of ${\mathrm{MoTe}}_{2}$ [185], as illustrated in Figure 6c. The heatmaps show the variation of the reaction grand potential for the transformation of the ${\mathrm{Te}}_{2}$‐terminated edge into four alternative configurations, as a function of pH and applied potential versus the SHE.

Another advantage of GC‐DFT is its ability to naturally account for the thermodynamic contributions of both charge‐transfer and ion–electron coupling processes. Quantities such as adsorption energies, interfacial capacitance, and coverage–potential relationships can be directly obtained as derivatives of the grand potential with respect to electrode potential or ion concentration. This stands in contrast to canonical DFT, where such effects are typically introduced through post hoc corrections that mix electrostatic and chemical contributions, and fail to reliably capture structural or spectroscopic trends under varying conditions [186].

Challenges remain in the widespread adoption of GC‐DFT. Convergence with respect to the fluctuating electron number can be unstable, especially when combined with complex solvation models or large‐scale periodic systems. The choice of reference potentials, treatment of the long‐range electrostatics, and the definition of the absolute potential scale must also be handled with care.

Despite these challenges, GC‐DFT represents a significant advance toward chemically and physically consistent modeling of electrochemical interfaces. It provides a direct route to computing thermodynamic properties at fixed potential, including surface charge, differential capacitance, and electrosorption behavior. As algorithms mature and become more widely available, GC‐DFT can be expected to become the standard tool for modeling electrocatalytic systems under realistic operating conditions.

### 4.3 Comparison of Constant‐Charge and Constant‐Potential Approaches

Although the constant‐charge and constant‐potential approaches impose different boundary conditions, with the former fixing the number of electrons and the latter fixing the electrode potential, they are fully consistent with each other when properly formulated. As demonstrated rigorously in refs. [187, 188], both approaches yield identical differential free energies in the thermodynamic limit, provided that the electrochemical degrees of freedom are treated consistently.

In the constant‐charge approach, a PCET reaction is described by the Gibbs free energy change

(37) $$\Delta G=\frac{dG}{dN_{H}}-\left({\tilde{\mu }}_{H^{+}}+{\tilde{\mu }}_{e}\right)$$

which approximates the change in system free energy G upon adding a hydrogen atom (i.e., a proton–electron couple). The electrochemical potentials ${\tilde{\mu }}_{e}$ and ${\tilde{\mu }}_{H^{+}}$ enter only as references of the energy change. This way, the dependence on the electrode potential appears only a posteriori, through the referencing of the computed $dG/dN_{H}$.

In the constant‐potential approach, the appropriate thermodynamic potential is the grand potential $\Omega (N_{H^{+}},{\tilde{\mu }}_{e})$, and the same process is described via

(38) $$\Delta \Omega ={\left.\frac{\partial \Omega }{\partial N_{H^{+}}}\right|}_{{\tilde{\mu }}_{e}}-{\tilde{\mu }}_{H^{+}}$$

where ${\tilde{\mu }}_{H^{+}}$ is introduced to reference the grand potential variation. In contrast to Equation (37), Equation (38) incorporates the dependence on the electrode potential directly through the explicit evaluation of $\partial \Omega /\partial N_{H^{+}}$ at constant ${\tilde{\mu }}_{e}$. Through the Legendre transformation, the derivative of the grand potential can be related to that of the canonical free energy as

(39) $${\left.\frac{\partial \Omega }{\partial N_{H^{+}}}\right|}_{{\tilde{\mu }}_{e}}={\left.\frac{dG}{dN_{H}}\right|}_{N_{e}^{\mathrm{net}}\left({\tilde{\mu }}_{e}\right)}-{\tilde{\mu }}_{e}$$

where $N_{e}^{\mathrm{net}}\left({\tilde{\mu }}_{e}\right)$ is the surface excess charge corresponding to the fixed potential ${\tilde{\mu }}_{e}$. Inserting Equation (39) into Equation (38) shows that ΔG=ΔΩ when canonical calculations are performed at constant $N_{e}^{\mathrm{net}}$ consistent with the GC description.

This result provides a formal proof that the two approaches are thermodynamically equivalent in the limit of infinite system size, resolving previous concerns about the validity of canonical schemes like the CHE. In particular, it confirms that computing reaction energies at fixed charge and introducing the electrode potential only in postprocessing does not introduce uncontrolled errors, as long as the same charge–potential relation is respected. This equivalence, however, assumes that all other physical approximations, such as solvation, interfacial structure, or electrostatic response, are treated consistently across both frameworks. For example, if electrode polarization is explicitly included in the GC‐DFT calculation, an analogous treatment must be incorporated into the CHE‐based analysis to ensure a meaningful comparison.

While the two approaches are formally equivalent, they differ in practice. GC‐DFT is conceptually cleaner for studying bias‐dependent processes: it avoids spurious potential shifts in small supercells, offers faster convergence with respect to system size, and yields direct access to quantities defined at fixed potential. However, it requires specialized implementations that allow control of the electronic chemical potential, and is more sensitive to modeling errors in solvation and interfacial capacitance. The accuracy of GC energetics critically depends on how well the potential–charge relation is captured.

Constant‐charge simulations, by contrast, are simpler to implement in standard DFT codes and more robust against errors in implicit solvent models and interfacial capacitance. They allow for flexibility in postprocessing: once the canonical energies are known, different electrochemical environments can be modeled by substituting alternative, e.g., experimental, charge–potential relations. On the other hand, as discussed in Section 4.1, constant‐charge calculations suffer from finite‐size artifacts due to the inability to maintain constant potential during electron transfer, which can lead to unphysical reaction energetics unless corrected through extrapolation schemes.

## 5 Reaction Kinetics

A major limitation of several computational approaches to electrochemical reactions, including thermochemical schemes such as the CHE, is the neglect of reaction kinetics. Within the CHE framework, it is typically assumed that protonation barriers are negligible, and that activation energies, if needed, can be inferred from correlations with the corresponding reaction step free energies. However, these assumptions are not universally valid. A predictive description of electrocatalytic processes requires the explicit evaluation of kinetic barriers, which involves a rigorous treatment of reaction pathways and transition states in a realistic electrochemical environment. Computing such barriers is crucial for modeling electrochemical reactivity and enables the prediction of experimentally accessible observables, including reaction rates, Tafel slopes, and product selectivity maps. The inclusion of kinetics is therefore essential for testing and validating theoretical models against experimental evidence.

### 5.1 Transition‐State Theory

In 1884, van't Hoff proposed that the rate constant, k, of a chemical process could be written as [189, 190]

(40) $$\frac{d\ln k}{dT}=\frac{E}{k_{B}T}$$

where E is an energy value characteristic of each reaction. If E is independent of T, integration of Equation (40) yields

(41) $$k=Ae^{-\frac{E}{k_{B}T}}$$

where A is the integration constant. In 1889, Arrhenius provided a physical explanation for the terms in Equation (41), which now bears his name [189, 191]. Arrhenius identified E as the activation energy required by the reactants to form products, while the prefactor A was treated as a constant of specific value for each chemical reaction.

Although the Arrhenius equation fits many empirical reaction rates, it offers limited mechanistic insight.

In 1935 Eyring, Polanyi, Evans, and Wigner independently developed transition‐state theory (TST) [192, 193, 194, 195].

It can be used to relate the rate constant to the potential energy surface (PES), a multidimensional function that specifies how the energy of a system of N particles depends to their 3N spatial coordinates. On the PES, stable species such as reactants, intermediates, and products correspond to regions around stationary points of minimum energy, since the probability of finding the atoms has a maximum there according to Boltzmann statistics. In contrast, the transformation of reactants into products passes through a region of low probability, typically over an energy ridge, but more generally a region of high free energy where low entropy can also play a role. This region is characterized by a dividing surface separating the reactant region on the energy surface from the rest of configuration space. When this dividing surface is given a small but finite thickness it is referred to as the transition state. A dip in the energy ridge corresponding to a ‘mountain pass’ is the most likely region for finding trajectories that take the system out of the reactant state.

In calculations of rate constants for transitions involving rearrangements of atoms in and on the surfaces of solids, such as chemical reactions and diffusion events, the harmonic approximation to TST [196] is most often used. There, the focus is placed on the point characterizing the mountain pass, the first order saddle point, where the gradient of potential energy with respect to atom coordinates is zero and the matrix of second derivatives, the Hessian, has one and only one negative eigenvalue. The eigenvector corresponding to the negative eigenvalue identifies the reaction coordinate at the transition state. We will refer to such first order saddle points as transition structures (TSs).

TST is formulated under four main assumptions [197]. First, the system stays in the ground electronic state throughout the reaction, i.e., the Born‐Oppenheimer approximation. Second, the motion of the atoms can be described with classical dynamics. Third, the reaction is assumed to be slow enough that the Boltzmann distribution is established in all degrees of freedom of the reactants up to and including the transition state. The fourth assumption is typically the weakest one. There it is assumed that a trajectory that makes it to the transition state and is heading away from the reactant region will proceed to products without recrossing the dividing surface. With these assumptions, an expression can be derived for the rate constant as [192, 198]

(42) $$k=\frac{k_{B}T}{h}\frac{q^{‡}}{\prod_{i}q_{i}}\;e^{-\frac{\Delta E^{‡}}{k_{B}T}}$$

where $q^{‡}$ is the partition function of the transition state, excluding the degree of freedom corresponding to the reaction coordinate, the $q_{i}$ are the partition functions of the reactants, h is Planck's constant and $\Delta E^{‡}$ is the activation energy. Within the harmonic approximation, the expression can be simplified to [196]

(43) $$k^{\text{HTST}}=\frac{\prod_{i}^{3N}\nu_{i,m}}{\prod_{i}^{3N-1}\nu_{i,s}}\;\exp\left\{-\left(E_{s}-E_{m}\right)/k_{B}T\right\}$$

where $E_{m}$ and $E_{s}$ are the values of the potential energy, and the $\nu_{i,m}$ and $\nu_{i,s}$ are the vibrational frequencies at the minimum and at the saddle point.

Alternatively, the rate constant can be expressed in terms of thermodynamic state functions, such as the Gibbs free energy of activation, $\Delta G^{‡}$, instead of partition functions [197]

(44) $$k=\frac{k_{B}T}{h}e^{-\frac{\Delta G^{‡}}{k_{B}T}}$$

Notably, TST is applicable to transitions involving a high enough free energy barrier when a transition state can be identified clearly. For example, it can be applied to the escape of gas molecules through a small hole on a container even though the process does not involve overcoming an energy barrier, only a free energy barrier due to small entropy in the hole, a natural choice for the transition state.

While conventional TST provides a framework for understanding chemical rates, it is important to remember its underlying assumptions and limitations. First, the neglect of recrossings of the transition state leads to an overestimation of the rate. To address this, a recrossing transmission coefficient can be applied to k [197]. Its value is necessarily between zero and one.

Moreover, since TST gives an overestimate of the rate constant, it is possible to variationally optimize the location and shape of the transition state [199]. The transition state dividing surface that gives largest free energy barrier gives the smallest estimate of the rate constant and thereby the most accurate TST rate estimate. In a formulation of variational TST the location of the transition state dividing surface is optimized [200]. This represents an optimization with respect to just one degree of freedom. More generally, the orientation as well as the location of the dividing surface can be optimized, an optimization with respect to 3N−2 degrees of freedom [201].

A question that is sometimes raised in connection with TST is the validity of separating the reaction coordinate from the other degrees of freedom [198, 202, 203]. This topic has been addressed by Miller and coworkers, in an attempt to introduce some non‐separability effects into the rate expression [204].

Finally, quantum tunneling might be significant, especially in the case of narrow energy barriers and/or particles of small mass such as protons. Then, a transition may occur even though the system does not possess enough energy to surmount the energy barrier $\Delta G^{‡}$. To express this, a tunneling transmission coefficient can be introduced in the rate constant [205, 206, 207]. More generally, the TST expression can be extended to include quantum statistical mechanics based on Feynman path integrals [208, 209]. Within a harmonic approximation, this reduces to instanton theory which allows for easier evaluation of the quantum mechanical rate constant, even directly from electronic structure calculations [210, 211].

Most applications of TST make use of the harmonic approximation where the key issue is finding the TSs on the PES describing the energetics of the system. Several numerical methods have been developed for this task and some of them are outlined in the following.

### 5.2 NEB and Dimer Methods

The algorithms that have been devised to locate TSs either assume both the initial and final states of the transition are known, and then the minimum‐energy path (MEP) connecting the reactant and product minima is found, or they rely only on a single starting point configuration of the atoms without information about possible product state(s) [197].

Among the path‐based methods, the nudged elastic band (NEB) approach is most widely used [212]. It requires as input the coordinates of the atoms at reactant and product energy minima, as well as a number of intermediate replicas of the system, referred to as ‘images’. These are subject to a force $F_{i}^{0}$

(45) $$F_{i}^{0}=-\nabla E{\left(R_{i}\right)}_{⊥}+F_{i}^{\left(s,∥\right)}$$

where $R_{i}$ specifies the atomic coordinates of image i and $-\nabla E{\left(R_{i}\right)}_{⊥}$ is the negative energy gradient in the directions orthogonal to the MEP. The images are relaxed perpendicular to the current path, while their distribution along the path, is controlled by a spring force $F_{i}^{\left(s,∥\right)}$

(46) $$F_{i}^{\left(s,∥\right)}=k\left(|R_{i+1}-R_{i}\left|-\right|R_{i}-R_{i-1}|\right){\hat{\tau }}_{i}$$

with k being the spring constant and ${\hat{\tau }}_{i}$ being an estimate of the tangent to the path at image i. The spring constant is assumed here for simplicity to be the same for all segments of the path, but can, for example, be chosen to increase with energy so as to give higher density of images near the barrier top [213].

An important issue is the way in which the local tangent, $\hat{\tau }$, to the current path is estimated at each image, especially when the parallel force acting on an image is large compared to the perpendicular ones. Improved numerical stability is obtained by approximating the tangent as the segment connecting the image to the adjacent one with higher energy [214].

In practical calculations, after convergence of the NEB calculation, the vibrations of the highest‐energy image are computed to verify whether it is a valid TS, namely, if it has a single negative eigenvalue of the Hessian.

Due to the fact that a practical calculation necessarily uses only a finite number of images, the resolution of the path is generally not high enough to place an image right at the TS.

This, however, can be accomplished in the climbing‐image NEB (CI‐NEB) algorithm [215]. There, the highest energy image is identified, possibly after a few regular NEB steps. Then, it is subject to a force which reverses the component of $-\nabla E(R_{i})$ along the path, without including the action of the spring

(47) $$F_{i}=-\nabla E\left(R_{i}\right)+2\left(\nabla E\left(R_{i}\right)\cdot {\hat{\tau }}_{i}\right){\hat{\tau }}_{i}$$

**FIGURE 7:**
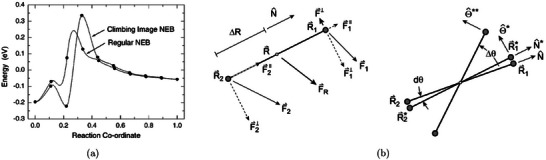
Saddle point search algorithms. (a) Energy profiles obtained via NEB and CI‐NEB for the CH_4_ dissociation to CH_3_ and H on an Ir(111) surface. The solid lines interpolate among different images (circles). Reproduced with permission [215]. Copyright 2000, American Institute of Physics. (b) Left panel: generation of the dimer at positions $R_{1}$ and $R_{2}$ with respect to the initial guess of TS, R, within the dimer algorithm. Right panel: rotation of the dimer along the lowest curvature mode at R. Reproduced with permission [216]. Copyright 1999, American Institute of Physics.

The other images are then required only to obtain a good enough estimate of the local tangent to the path. As a result of the application of Equation (47), the CI‐NEB raises the energy of the climbing image along the path while lowering it in the perpendicular directions. Thus, the images should all converge to the MEP and the highest one to the TS, as displayed in Figure 7a.

A more powerful but also more challenging approach is to avoid introducing any preconceived notion of product state(s) and converge on a TS from some starting point that may be close to the initial state minimum, or possibly a guess of the atom coordinates at a TS. The dimer method is an approach of this sort that is often used [216]. There, the initial atom coordinates are slightly displaced by a vector R, generating two images of the system $R_{1}$ and $R_{2}$ referred to as a dimer

(48) $$R_{1}=R_{\mathrm{guess}}+R$$

(49) $$R_{2}=R_{\mathrm{guess}}-R$$

as illustrated in the left panel of Figure 7b. Because there is no prior knowledge of the direction of R, this is chosen arbitrarily. Then, energy and the forces are evaluated at the two images, $R_{1}$ and $R_{2}$. Additionally, the curvature of the PES is computed as

(50) $$C=\frac{\left(E_{1}+E_{2}\right)-2E_{\mathrm{midpoint}}}{{\left(\Delta R\right)}^{2}}$$

It follows from Equation (50) that minimizing $E_{\mathrm{midpoint}}$ also implies the minimization of the curvature. Therefore, the dimer is rotated toward the direction of lowest curvature, i.e., the eigenvector corresponding to the lowest eigenvalue. The gradient of the potential energy acting on the center of the dimer is then inverted along this direction and it is moved in the direction opposite to the transformed gradient, effectively moving the system closer to the TS, see Figure 7b, right panel. The numerical procedures used to perform the dimer rotation and translation include accelerated steepest descent and conjugate gradient methods. Later, there have been several improvements of the algorithm [217, 218].

Even when a product state is specified and the (CI‐)NEB used to find the MEP, it is advantageous to interrupt the calculation when it is clear whether any intermediates exist between reactants and products and complete the TS search using the dimer method starting with the image of highest energy. This strategy saves computational effort compared to the convergence of a full NEB or CI‐NEB simulation to a tight tolerance, especially for large systems. These algorithms are implemented in common electronic structure software packages as Quantum ESPRESSO [47, 48], VASP [46], CP2K [53], GPAW [54], NWCHEM [219], ORCA [213], and in the python module ASE [220].

### 5.3 Kinetic Barriers From MD and Enhanced Sampling

Activation energies for reaction steps can also be estimated using MD simulations, whose trajectories must explore all possible pathways connecting reactants and products. This approach offers insight into the ensemble of transition states that separates them. In contrast to static methods (see Section 5.2), which probe the PES, MD directly accesses the free energy surface (FES). While the PES includes only potential energy contributions based on interatomic interactions, the FES incorporates both potential and entropic effects, thus providing a more complete thermodynamic description of the system [221].

Typical electrocatalytic reactions occur on timescales ranging from microseconds to milliseconds [222], which lie far beyond the reach of conventional MD simulations. AIMD typically accesses timescales of a few tens of picoseconds, whereas classical or machine learning‐accelerated MD can extend to several tens of nanoseconds (see also Section 6.4). As a consequence, a simple MD trajectory starting from a metastable state *A* will remain trapped in that basin for the entire simulation and will not overcome the free energy barriers necessary to reach a second metastable state *B*. Even in rare cases where transitions are observed, their number is insufficient to provide statistically meaningful results. To address this issue, MD must be combined with enhanced sampling techniques specifically designed to study such rare events.

For a comprehensive discussion of enhanced sampling techniques, we refer the reader to dedicated reviews on this topic, such as refs. [223, 224]. Here, we focus on the most widely used family of methods in electrocatalysis, which rely on the introduction of collective variables (CVs). A CV is defined as a coarse‐grained coordinate capable of distinguishing between relevant metastable states. Enhanced sampling methods based on CVs apply an external bias (entirely distinct from the electrical bias that can be applied to control the electrode potential) to promote fluctuations along the CVs and thereby reduce the free energy barriers that hinder sampling [224].

In practice, a set of CVs s(R) is expressed as a group of functions depending on the set of atomic coordinates {R}. In this framework, it is generally advantageous to study the FES as a function of s rather than directly in terms of {R}. The FES F(s) is then defined statistically, under the assumption of ergodicity, as

(51) $$F\left(s\right)=-\frac{1}{\beta }\log\left[P\left(s\right)\right]\approx -\frac{1}{\beta }\lim_{t\to \infty }\log\left[N\left(s,t\right)\right]$$

where $\beta =1/k_{B}T$, P(s) is the equilibrium distribution of the CVs, and N(s,t) is the normalized histogram obtained from sampling the MD trajectory. Once F(s) is known, the free energy difference between any two metastable states *A* and *B* can be computed as

(52) $$\Delta F_{A,B}=-\frac{1}{\beta }\log\frac{\int_{A}ds\;e^{-\beta F(s)}}{\int_{B}ds\;e^{-\beta F(s)}}$$

**FIGURE 8:**
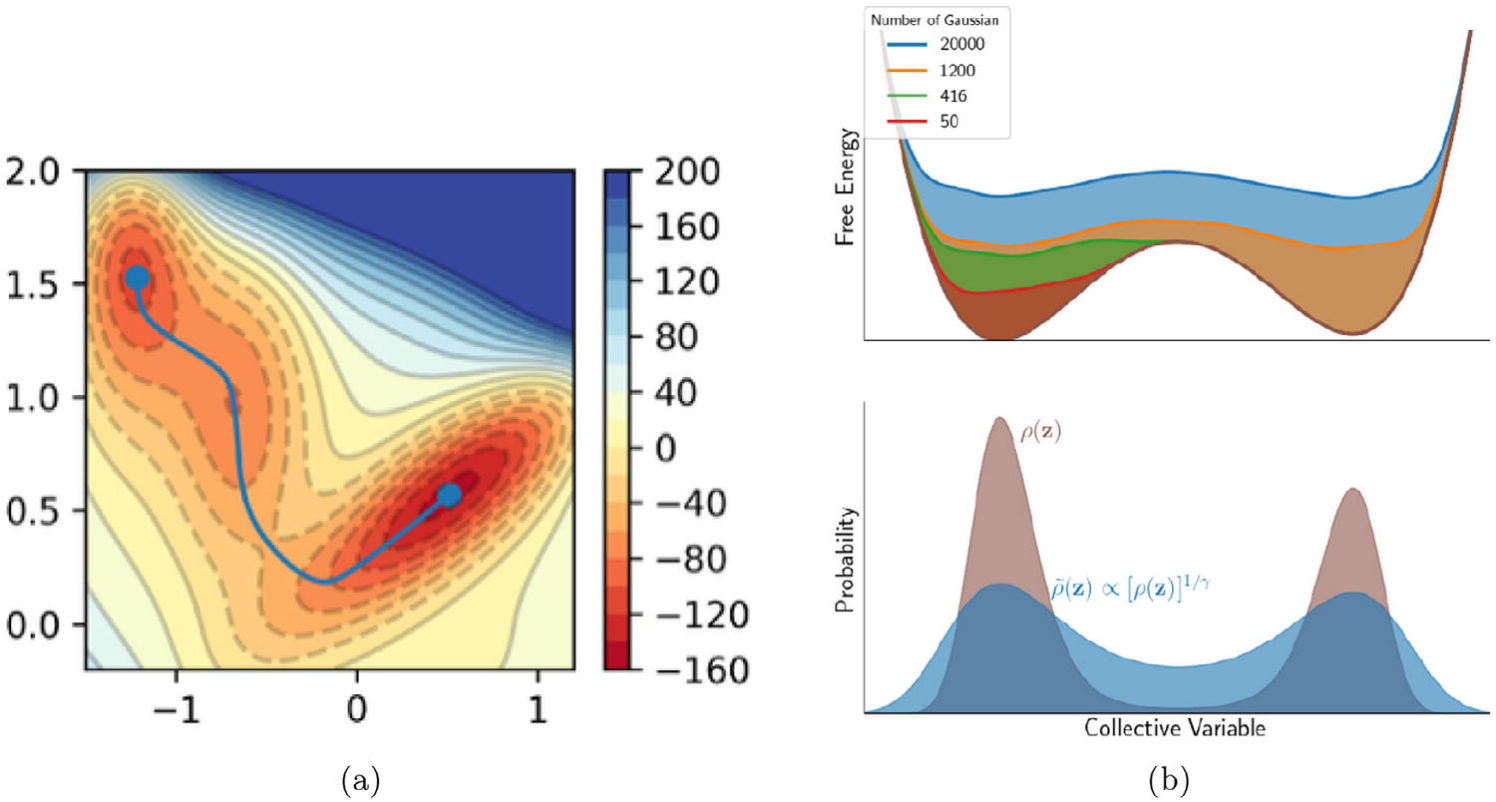
Enhanced sampling techniques. (a) Example of FES in a 2D space of generic CVs. The path between the basins of two main states is shown in blue. The colorbar identifies the free energy scale of the regions of the FES. Reproduced under the terms of the CC‐BY‐NC‐ND 3.0 license [225]. Copyright 2024, The Authors, published by Wiley Periodicals LLC. (b) Sketch of the effects of the bias on the FES (upper panel) and on the probability distribution (lower panel) in a well‐tempered metadynamic simulations. Upper panel: the two basins of the FES are progressively filled by the deposited gaussian bias along the biased trajectory. The biased FES is progressively barrierless, allowing a uniform sampling of the whole CV range. Lower panel: the probability distribution of the biased simulation (light blue) is smoother with respect the original distribution (dark pink), resulting in an enhanced possibility of sampling the TS region between the two basins. Reproduced with permission [223]. Copyright 2022, CC BY 4.0, The Authors, published by the University of Colorado Boulder.

Once the full FES is available, the minimum free energy path connecting the states can also be identified, making it possible to extract all the barriers that separate the states. An illustrative example of a hypothetical two‐dimensional FES is shown in Figure 8a, where multiple basins and the connecting path between two main states can be clearly distinguished [225].

Accurate estimation of the FES and the free energy difference $\Delta F_{A,B}$ requires uniform sampling of N(s,t) over the relevant CV space within a finite simulation time. This is hindered by the presence of free energy barriers. To overcome this limitation, a bias potential V(s) can be introduced. This potential depends on the CVs and is designed to enhance sampling in poorly visited regions. The MD trajectory then explores a biased FES defined as $F_{V}\left(s\right)=F\left(s\right)+V\left(s\right)$. The original unbiased FES can be reconstructed through a reweighting procedure (strictly valid only if the bias potential is static), as

(53) $$F\left(s\right)=-\frac{1}{\beta }\log\left[N_{V}\left(s\right)\right]-V\left(s\right)$$

where $N_{V}\left(s\right)$ is the histogram collected during the biased MD trajectory in which the barriers have been artificially lowered [224]. A schematic overview of the connection between the FES, the equilibrium distribution of CVs, and their modification under biased sampling is shown in Figure 8b, where the progressive flattening of the FES by the applied bias (upper panel) leads to a smoother, more uniform CV distribution (lower panel), enhancing the sampling of the TS region.

Various enhanced sampling methods are based on these general principles, differing in how the bias potential is constructed. In an extreme limit, V(s) should be equal to −F(s), which would lead to a uniform distribution $N_{V}\left(s\right)$ across the CV space and complete flattening of the energy barriers. Practically, V(s) is constructed to lead to a smoother version of N(s), without completely smoothing out the energy barriers to avoid sampling highly unphysical regions. In all the cases, since F(s) is not known in advance, different strategies have been developed to approximate this condition. We do not provide detailed descriptions of each method but refer interested readers to the following common approaches: umbrella sampling [226], metadynamics [227] and its well‐tempered variant [228], and on‐the‐fly probability enhanced sampling (OPES) in its various formulations [229, 230].

Regardless of the specific enhanced sampling algorithm employed, the choice of CVs is central to the success of the simulation. The efficiency of exploration and the convergence of the FES depend critically on the quality of the biased CVs. Ideally, a well‐designed CV should clearly separate the relevant metastable basins and include all the slow modes, i.e., the degrees of freedom that evolve on long timescales and are therefore the most difficult to sample [224, 231].

If an important slow mode is omitted or a poorly discriminating CV is chosen, convergence on the FES becomes inefficient. In metadynamics, for instance, this typically results in hysteresis cycles, with the trajectory repeatedly jumping between basins without adequately sampling the transition region. Biasing multiple CVs can help capture all relevant modes, but this also results in increased computational cost. In practice, the use of more than three or four biased CVs becomes unmanageable. Choosing optimal CVs is thus a delicate task that requires both physical intuition and system‐specific expertise [224].

In many applications, especially for reactions involving small molecules on catalytic surfaces, simple geometrical CVs are often considered. These include atomic distances, differences between distances, and coordination numbers. For example, in the Volmer step of HER, proton transfer from a water molecule to the surface can be modeled by biasing the difference between the O–H bond distance and the distance between the same hydrogen atom and the surface [232]. Similarly, in the Heyrovsky and Tafel steps of HER, the H–H distance is commonly used as a CV [233].

More complex reactions may require composite CVs. These can include, for instance, the generalized coordination number involving O, H, and surface atoms for water dissociation on oxide surfaces [234], or combinations of O, H, and C distances in the context of ${\mathrm{CO}}_{2}\mathrm{RR}$ [118].

Despite their intuitive appeal, geometrical CVs must be used with care. They can drive the system into unphysical and/or unwanted regions of configuration space. For example, the CV proposed for the HER Volmer step must be used alongside a constraint on the water–surface distance; otherwise, the bias may push the water molecule away from the interface. Moreover, a poorly chosen CV can drive the reaction along an unnatural pathway, leading to an inaccurate estimation of the activation barrier due to a negative influence introduced by the CV choice.

For these reasons, the development of improved CVs remains an active area of research, to which data‐driven strategies based on machine learning have been added in recent years [235].

### 5.4 Combined Thermodynamic and Kinetic Studies

A comprehensive understanding of electrochemical reactivity requires theoretical frameworks that systematically account for both thermodynamic driving forces and kinetic feasibility. Historically, computational catalyst screening and mechanistic studies have leaned heavily on thermodynamic descriptors, most notably, adsorption free energies, and limiting potentials derived via the CHE model (Section 2). While this approach has facilitated broad insights into trends in activity and selectivity, its inherent neglect of kinetic barriers has led, in many cases, to incomplete or misleading mechanistic interpretations.

Increasingly, it has become clear that thermodynamic analyses alone cannot reliably predict reactivity or product distributions. For instance, studies comparing thermochemical models with more detailed kinetic analyses in ${\mathrm{CO}}_{2}\mathrm{RR}$ revealed that neglecting electric field effects and potential‐dependent kinetics can lead to misinterpretation of key trends [111].

Similarly, investigations into the ORR demonstrated that neither the thermodynamic stability of intermediates nor the limiting potential alone could explain the experimentally observed selectivity between two‐ and four‐electron pathways. These findings underscore that thermodynamic stability must be complemented by a detailed kinetic perspective [236].

**FIGURE 9:**
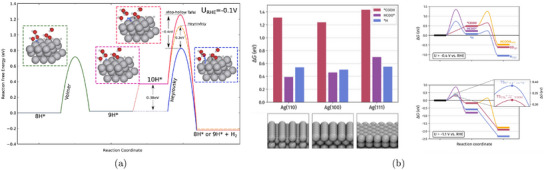
Combined thermodynamic and kinetic studies. (a) Grand‐canonical free energy diagram of HER on Pt(111) at U=−0.1 V versus RHE, illustrating the Volmer–Tafel and Volmer–Heyrovsky reaction pathways with one monolayer $H^{*}$ coverage. Insets depict representative transition states: the Volmer step involves a proton approaching an atop site before relaxing into a hollow site; the Heyrovsky step shows a hollow‐bound $H^{*}$ moving toward an atop site while associating with an incoming proton. The Tafel step features the association of an atop‐bound $H^{*}$ with a neighboring hollow‐bound $H^{*}$. Reproduced with permission [237]. Copyright 2020, American Chemical Society. (b) Left panel: free energies of adsorption of *COOH, *H and HCOO* on silver surfaces Ag(110), Ag(100), and Ag(111). Right panel: full reaction grand‐potential profiles with kinetic barriers at different applied potentials U=−0.4 V (above) and U=−1.1 V versus RHE (below). Reproduced with permission [238]. Copyright 2024, American Chemical Society.

Kinetics alone is likewise insufficient. While activation barriers provide crucial insight into reaction rates, they say little about the thermodynamic feasibility of forming or sustaining the required intermediates under electrochemical conditions. This has been exemplified in HER, where water‐assisted proton transfer pathways on platinum surfaces were shown to be kinetically favorable only when the reorganization of the solvent environment and the thermodynamic stability of intermediates were simultaneously considered [237], Figure 9a. Ignoring these factors can lead to incorrect identification of dominant mechanistic routes.

Taken together, these observations motivate the development and adoption of unified kinetic–thermodynamic frameworks capable of capturing the complex interplay between energetics and kinetics in electrochemical systems. In the case of ORR, such a framework has proven essential to rationalize product selectivity, which does not emerge from thermodynamic or kinetic control alone, but from a delicate balance between intermediate stability and activation barriers [236]. As the field advances toward predictive modeling of complex electrocatalytic networks, such integrated approaches will be indispensable.

In the field of electrochemical ${\mathrm{CO}}_{2}\mathrm{RR}$, where multiple competing products (e.g., CO, HCOOH, ${\mathrm{CH}}_{4}$, $C_{2}H$ 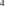, and alcohols) can form, the need for a combined kinetic–thermodynamic approach is even more pronounced and it is essential to explain product distributions on silver and copper catalysts [80]. It has also been shown that reaction paths that show the most favorable thermodynamics, such as the one leading from ${\mathrm{CO}}_{2}$ to HCOO^−^ through the HCOO* intermediate [238], see Figure 9b, are not necessarily the overall favored reaction path. Indeed, when combining the study of kinetic barriers to thermodynamics, it appears that the production of the stable intermediate HCOO* is hindered by a very large kinetic barrier, as shown in the right panel of Figure 9b. Instead, the final reaction product is determined by a delicate interplay between the thermodynamic stabilization of the *H and *COOH intermediates and the corresponding kinetic barriers, as reported in the right panel of Figure 9b. Here, a change in applied potential from ─0.4 V to above ─1 V versus RHE determines a dramatic switch in product selectivity, that passes from a dominant HER to CO production.

The importance of reaction kinetics remains critical to determine which pathways are operative under realistic electrochemical conditions [236]. In investigations of $C_{2}$ product formation on Cu, it was demonstrated that accounting for both the pH‐dependent thermodynamics and multistep kinetics was essential to explain the potential‐dependent production of ethylene [239].

A striking example of voltage‐dependent kinetic control was reported in the context of the selectivity between CO and HCOOH on Cu, a phenomenon that cannot be understood from free energies alone [240]. Constant‐potential analysis revealed that the apparent preference for one product over another emerges only after considering the interplay of activation barriers and surface coverages under applied bias. These insights are further reinforced by recent studies [88, 241], which emphasize that advanced modeling of electrochemical systems must go beyond traditional CHE‐based thermodynamics.

These studies support hybrid frameworks that combine potential‐dependent free energy profiles, explicit or implicit solvation, barrier calculations, Brønsted‐Evans‐Polanyi relations (see Section 6), and microkinetic simulations (see Section 5.5). Such models are capable of incorporating realistic interfacial structures, solvent organization, and electric fields, thereby combining theory and experimental conditions.

Taken together, these studies prove that neither thermodynamics nor kinetics alone are always sufficient to understand or predict electrochemical behavior. Only through an integrated treatment, where thermodynamic stability defines the accessible reaction space and kinetic modeling identifies the dynamically favored pathways, can theory provide a predictive and mechanistically accurate description of electrochemical transformations.

### 5.5 Microkinetic Modeling

Recognizing the importance of a combined treatment of reaction thermodynamics and kinetics, microkinetic modeling aims at providing a comprehensive framework to describe catalytic processes through networks of elementary steps connecting reactants to products. The activation energies computed using either TST (Section 5.2), or enhanced sampling in MD (Section 5.3), can be used to estimate elementary reaction rates, which serve as input for microkinetic modeling. This step provides the essential connection between atomistic simulations and the overall catalytic performance of a system, enabling the prediction of observable quantities such as turnover frequencies and product selectivities under realistic operating conditions, potentially guiding the design of new catalysts with improved performance.

In practice, the derivation of a microkinetic model begins with identifying the species deemed to participate in a reaction process and the plausible elementary steps between them [242]. Next, the equilibrium constants of each reaction step are derived from the Gibbs energies of the intermediates computed with DFT, while rate constants are obtained, for example, via TST (see Section 5.1) [242]. According to the law of mass action, the rate $r_{j}$ for the elementary reaction step j can be expressed as

(54) $$r_{j}=k_{f}\prod_{\begin{array}{c} i\;\in \;\text{reactants} \end{array}}a_{i}^{-n_{ij}}-k_{b}\prod_{\begin{array}{c} i\;\in \;\text{products} \end{array}}a_{i}^{n_{ij}}$$

where $k_{f}$ and $k_{b}$ are the forward and backward rate constants, $a_{i}$ is the activity of species i and $n_{ij}$ is the stoichiometric coefficient of species i, with $n_{ij}<0$ if i is a reactant and $n_{ij}>0$ if it is a product [242, 243, 244, 245]. Because in principle the identity of the rate determining steps is unknown a priori, microkinetic analysis makes no assumption on their nature, evaluating the whole reaction network [242]. The time evolution of a species concentration is then expressed as a function of the rates of the elementary steps in which it participates. For heterogeneous processes, this corresponds to describing the evolution in time of the fractional coverage $\theta_{i}$ of the catalyst surface by species i, as [242]

(55) $$\frac{d\theta_{i}}{dt}=\sum_{j}n_{ij}r_{j}$$

where $n_{ij}$ is the stoichiometric coefficient (with sign) of species i in reaction step j: $n_{ij}<0$ if i is consumed as a reactant, and $n_{ij}>0$ if it is produced. The sum of the surface coverages of all species, including that of free sites, is equal to one due to site number conservation. Under steady‐state conditions, concentrations and surface coverages of all species remain constant in time, and the rates of formation and consumption are balanced [242]. In this case, the set of differential equations reduces to a system of coupled algebraic equations that can be solved straightforwardly.

**FIGURE 10:**
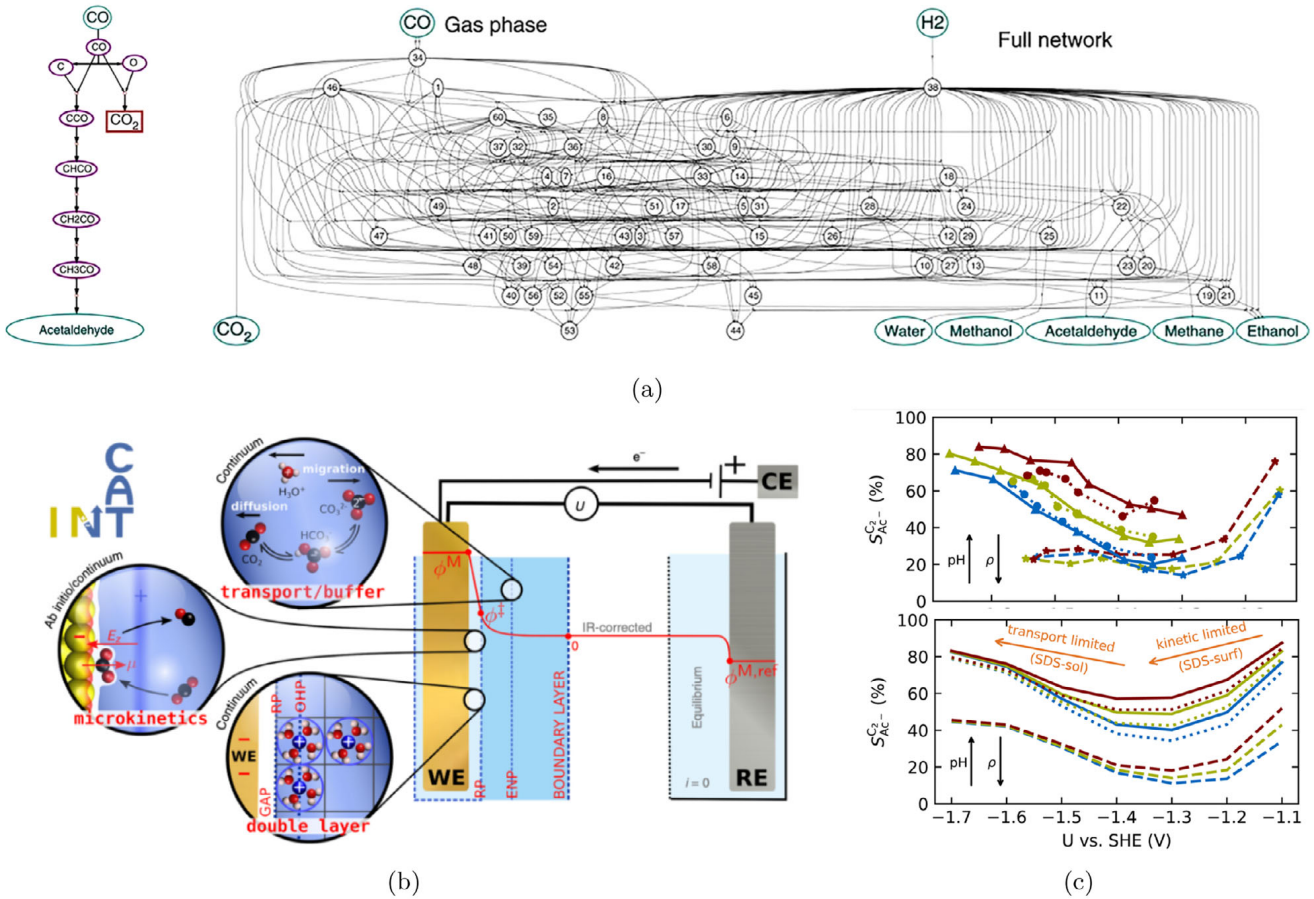
Microkinetic modelling. (a) Left panel: relevant elementary reactions for acetaldehyde production from syngas on Rh(111). Right panel: schematic illustration of all possible networks between elementary steps in the conversion of syngas to CO_2_, H_2_O, CH_4_, CH_3_OH, acetaldehyde, and ethanol. Reproduced under the terms of the CC‐BY 4.0 license [247]. Copyright 2017, The Authors, published by Springer Nature. (b) Schematic representation of the microkinetic and mass transport models implemented in the CatINT package. The scheme explicitly describes the double layer at the cathode, including the presence of a gap capacitance at the outer Helmholtz plane. Reproduced under the terms of the CC‐BY 4.0 license [246]. Copyright 2020, The Authors, published by Springer Nature. (c) Experimental (upper panel) and theoretical (lower panel) dependence on applied potential versus SHE of the selectivity of Cu toward acetate production with respect to all other C_2_ products. The different colors represent varying electrolyte pH (13.7 blue, 14.0 green, 14.3 dark‐red) and the solid, dotted, and dashed lines mark experiments and simulations with varying surface roughness ρ=10,13,65, respectively. Reproduced under the terms of the CC‐BY 3.0 license [248]. Copyright 2022, The Authors, published by the Royal Society of Chemistry.

To capture experimental catalytic performances, this microkinetic approach is usually coupled with a mass transport model to describe the migration of reactants and products toward and away from the catalyst, respectively. Ideally, in electrochemical systems, this should also incorporate the effects of the double layer, ion–ion interactions, buffer equilibria and the interactions of the electrolyte with the electrode, since all these factors influence the local reaction environment and therefore the observed kinetics [246]. A software package devised to this purpose is Catalysis at the Interfacial Node Transport (CatINT) [246], which employs a 1D generalized modified Poisson–Nernst–Planck approach to model mass transport and is schematized in Figure 10b. In this framework, the Nernst–Planck and Poisson equations are used to describe the transport of ionic species in the electrolyte and the corresponding electrostatic potential, respectively.

Mass transport is coupled with the microkinetic model at the reaction plane, which is identified with the outer Helmholtz plane. This interface is characterized by a gap capacitance that accounts for repulsive interactions at the boundary between surface and water, as illustrated in Figure 10b.

A central challenge in microkinetic modelling is the identification of all possible interconnections between reactions and, in a practical sense, a balance must be sought between the computational cost of estimating all kinetic parameters and the identification of the dominant elementary steps. For example, the left panel of Figure 10a illustrates the subset of elementary steps identified as relevant for acetaldehyde formation on Rh(111) from syngas, whereas the right panel of Figure 10a depicts the complete network of all possible pathways involved in syngas conversion to CO_2_, H_2_O, CH_4_, CH_3_OH, acetaldehyde and ethanol [242]. Notably, such network comprises over two thousand distinct pathways. To simplify this task, softwares like MECHEM [249], Rule Input Network Generator (RING) [250], and Reaction Mechanism Generator (RMG) [251] can automatically devise reaction networks [242]. Alternatively, an iterative approach can be adopted, wherein a reaction mechanism is first proposed using estimated parameters [242]. Then, the most relevant steps are identified, and the model is refined accordingly. Furthermore, experimental evidence can aid in uncovering reaction mechanisms and validating theoretical results [252]. For instance, pH and kinetic isotope effects can indicate the involvement of protons or specific atoms in rate determining steps, while operando techniques like in‐situ spectroscopies can confirm the presence of key intermediates and surface coverages [246, 252]. Additionally, in electrocatalysis, Tafel slopes can represent an interesting parameter of comparison between theory and experiments [253, 254]. More in detail, they express how much the applied potential has to change (ΔU) for the reaction rate to increase by an order of magnitude as

(56) $$\Delta U=a+b\log_{10}i$$

where b is the Tafel slope and i is the current. From a mechanistic perspective, Tafel slopes can be used to identify the nature of the rate determining step, provided certain conditions are met: no mass transport limitations, no changes in ohmic resistance with potential, no significant reverse reaction, constant temperature, and constant number of available catalytic sites [254]. Typical Tafel slopes have been classified in the literature for the rate determining step in electrochemical CO_2_RR. For example, the identity of possible rate limiting steps in CO production has been linked to specific values of the Tafel slope [255]. For the initial PCET or single electron transfer to CO_2_, a Tafel slope of 118 mV dec^−1^ was reported. In contrast, for intermediate proton transfers and subsequent PCETs or electron transfers, the authors calculated Tafel slopes of 59 and 39 mV dec^−1^, respectively. Lastly, a Tafel slope of 30 mV dec^−1^ was obtained for CO desorption.

A representative example of joint experimental and theoretical study sought to reveal the rate determining step of the CO_2_RR on Au electrodes between *CO_2_ adsorption, *CO_2_ hydrogenation to *COOH and *COOH hydrogenation to *CO [246]. Experiments indicated that the overall rate was independent from pH, suggesting that protons were not involved in the rate determining step, consistent with previous measurements of kinetic isotope effects. DFT calculations of the CO_2_RR mechanism were then performed on a stepped (211) Au surface, involving a sequence of PCETs following *CO_2_ adsorption, and estimating the density of active sites from the interatomic distances on the (211) steps. Based on these simulations, a microkinetic and mass transport model was constructed, and the degree of rate control, $X_{\mathrm{RC}}$, was evaluated for *CO_2_ adsorption and *COOH formation. $X_{\mathrm{RC}}$ quantifies the sensitivity of the reaction rate to the variation in the energy of a specific TS, and is defined as

(57) $$X_{\mathrm{RC},i}={\left.\frac{d\ln r}{d\ln k_{i}}\right|}_{\begin{array}{c} K_{i,\mathrm{eq}}\\ k_{j\neq i} \end{array}}$$

where r is the reaction rate, $k_{i}$ the rate constant of step i, and all other parameters are kept unchanged ($K_{i,\mathrm{eq}}$, $k_{j\neq i}$) [242]. In agreement with experiments, the model showed that at moderate and high overpotentials *CO_2_ adsorption was rate limiting, and no protons were involved. Importantly, such mechanistic insights derived from the microkinetic model extend beyond the information given by individual reaction profiles computed with DFT.

Microkinetic analysis also provides access to the electrocatalyst selectivity for a given product i, defined as [242]

(58) $$S_{i}=\frac{r_{i}}{\sum_{j}r_{j}}$$

where the denominator sums the rates of all competing products, or a selected subset if a relative selectivity is desired. Product selectivity varies strongly with the applied electrode potential, as illustrated in Figure 10c.

Here, the potential dependence of the selectivity of Cu toward acetate among C_2_ products, $S_{{\mathrm{Ac}}^{-}}^{C_{2}}$, is determined using a coupled microkinetic–transport model [248]. This shows that $S_{{\mathrm{Ac}}^{-}}^{C_{2}}$ depends on local pH, catalyst roughness, and applied potential. At low overpotentials, the protonation of adsorbed ketene (H_2_CCO*) is slow, favoring acetate formation through a potential‐independent solution reaction. As the overpotential becomes more negative, the surface reduction pathway accelerates, decreasing acetate selectivity. At very negative potentials, the rise of local ${\mathrm{OH}}^{-}$ concentration due to higher current densities again promotes the solution‐phase reaction, producing the characteristic U‐shaped selectivity versus potential observed experimentally (see upper panel of Figure 10(c)).

## 6 Reaction Descriptors and Machine Learning Methods

The previous Sections have shown that first‐principles simulations can provide a reliable framework for describing the elementary steps of electrocatalytic reactions. However, their application to large‐scale catalyst screening and complex electrochemical environments is often hindered by the high computational cost and short accessible timescales. To address these limitations, reaction descriptors have been introduced. These are simple variables that retain essential chemical information and correlate with more complex properties such as adsorption or activation energies. Descriptors enable systematic comparison across materials and help reduce both the complexity and the number of required simulations. An example is provided by scaling relations, which express approximate linear correlations between the energies of related adsorbed species or reaction steps. Such relations allow unknown values to be estimated from a reduced set of reference calculations. Building on the descriptor approach, machine‐learning (ML) techniques have been developed to predict adsorption and reaction energies from structural or electronic features. Moreover, ML methods are now also employed to construct ML‐based force fields that approach the accuracy of ab initio simulations while dramatically lowering computational cost.

### 6.1 Scaling Relations

A full kinetic description of electrochemical processes requires knowledge of both reaction energies and the corresponding activation barriers. This complexity poses a considerable challenge for high‐throughput catalyst screening, particularly in the case of multi‐electron reactions. To reduce the dimensionality of the problem and the number of explicit calculations, various scaling relations have been identified that link key energetic parameters [243].

One foundational insight originates from molecular adsorption. In the case of physisorption, the binding strength correlates with adsorbate size. For example, the adsorption energy of *n*‐alkanes increases linearly with chain length across a variety of surfaces, including Pt(111), graphite, and MgO [256]. For chemisorption, species that bind through the same element exhibit linearly correlated adsorption energies. A notable case is the adsorption of hydrogen‐containing molecules of the form ${\mathrm{AH}}_{x}$ (where A = C, N, O, S), which are linearly related to the adsorption energy of atom A [257]. The relationship takes the form

(59) $$\Delta E^{{\text{AH}}_{x}}=\gamma \left(x\right)\;\Delta E^{A}+\xi$$

where the slope γ(x) is independent of the metal surface. Based on bond order conservation, if atom A has a maximum valence $x_{\max}$, and $x\leq x_{\max}$ of these bonds are saturated by bonding to other atoms, then the scaling slope is given by

(60) $$\gamma \left(x\right)=\frac{x_{\max}-x}{x_{\max}}$$

**FIGURE 11:**
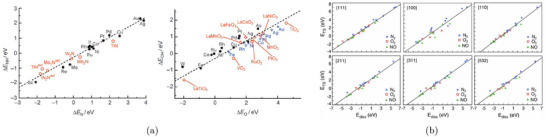
Scaling relations in catalysis. (a) Calculated adsorption energies of ${\mathrm{NH}}_{x}$ and OH on transition metals and metal compounds plotted as a function of the adsorption energies of N and O, respectively. Reproduced with permission [258]. Copyright 2008, WILEY‐VCH Verlag GmbH & Co. (b) The linear transition state scaling relations for $N_{2}$, $O_{2}$, and NO dissociation over six different transition‐metal surface facets as a function of the dissociation energy. Reproduced with permission [265]. Copyright 2013, Springer Science+Business Media.

The intercept ξ in Equation (59) can be obtained from experimental measurement or theoretical calculations on a single reference system, enabling scaling across a wide range of catalysts, as shown in Figure 11a. The adsorption energies of ${\mathrm{NH}}_{x}$ and OH show a perfect linear scaling with respect to the adsorption energy of the N and O atoms on various transition metals, metal nitrides and oxides [258].

Beyond their original formulation from the analysis of A/${\mathrm{AH}}_{x}$ adsorbates, adsorption‐energy scaling relations can be placed on a more general theoretical footing. Linear relations between adsorption energies are obtained only when both quantities depend in a proportional manner on the same underlying electronic variables describing the adsorption [259]. The adsorption energies of two species can be written as $\Delta E_{1}=f\left(\left\{\omega_{i}\right\}\right)+\alpha_{0}$ and $\Delta E_{2}=g\left(\left\{\omega_{i}\right\}\right)+\beta_{0}$, where $\omega_{i}$ denotes a set of parameters that characterize the electronic structure of the surface–adsorbate system. These parameters may, for example, represent the group numbers or valence electron counts of the adsorbate and of the atoms constituting the surface active site, or the work function, the excess charge, the d‐band center or other electronic properties [260]. Simple linear scaling is obtained only if f and g are proportional functions of a common variable; otherwise, deviations from scaling arise in a systematic manner. By explicitly expressing $\Delta E_{1}$ and $\Delta E_{2}$ as smooth functions of their respective variables, deviations from linear scaling can be parameterized, even though a universal scaling relation with a constant offset is not restored.

The scaling of the reaction energy of an elementary step can be further generalized as

(61) $$\Delta E=\sum_{i=1}^{N}\Delta \gamma_{i}\;\Delta E^{A_{i}}+\Delta \xi$$

where the sum runs over all surface‐bound atoms $A_{i}$, with $\Delta \gamma_{i}$ capturing the change in bond order upon reaction. Such relations have proven applicable not only to hydrogenated species but also to $C_{2}$ hydrocarbons [261], and they extend beyond pure metals to metal‐terminated compounds such as oxides, nitrides, sulfides, and carbides [258, 262, 263, 264].

A second, widely adopted scaling concept relates activation energies to reaction energies. This is the essence of the Brønsted–Evans–Polanyi (BEP) relation [266, 267]. BEP relations describe linear dependencies between activation and reaction energies, while more general TS scaling relations express the TS energy as a linear combination of adsorption energies as [243, 268]

(62) $$E^{\mathrm{TS}}=\sum_{i}\gamma_{i}\Delta E_{i}+\xi$$

here, $E^{\mathrm{TS}}$ is the TS energy of a reaction step on a catalyst and $\Delta E_{i}$ are adsorption energies of relevant surface bound species in the reaction. Equation (62) provides a linear map between TS energies and adsorption energies which has been proved to hold for small energy variations. For larger ranges, the relation deviates from linearity. As shown in Figure 11(b), TS scaling for $N_{2}$, $O_{2}$, and NO dissociation has been systematically mapped across stepped, kinked, close‐packed, and open metal surfaces, with the dissociation barrier scaling linearly with the reaction energy [265]. These relations typically have slopes near unity, suggesting that the TS closely resembles the final state in electronic structure. Structural effects are reflected in vertical offsets between scaling lines, where close‐packed surfaces are generally less reactive than under‐coordinated ones such as steps or kinks.

The universality of such relations extends across a broad class of reactions. Linear BEP‐type correlations have been observed for the dissociation of CO, NO, $N_{2}$, and $O_{2}$ [269], as well as for coupling reactions involving C–C, C–N, C–O, N–O, and O–O bonds [268]. The slope of the scaling relation typically ranges between 0.5 and 1.0, depending on the reaction pathway and character of the TS. Simple dissociation reactions often yield slopes near 1, while PCET reactions tend toward 0.5, consistent with their typical transfer coefficients [77]. While these trends are most robust for metal surfaces, deviations are known to occur on metal oxides [270], and notable exceptions arise for systems with unusually weak bonds, such as $H_{2}$ dissociation [243].

The parameters of scaling relations depend sensitively on the local structure of the active site. In particular, coordination and ligand effects have been identified as key factors controlling both the existence of scaling relations and the value of their offsets. Variations in coordination number modify the balance between metal–adsorbate bonding and charge redistribution, leading to systematic shifts in adsorption energies that are reflected in the offsets of scaling relations [271]. More generally, changes in local coordination and ligand environment can determine whether two adsorbates obey a linear scaling relation or deviate from it, indicating that scalability is a site‐dependent property rather than an intrinsic feature of a given adsorbate pair [272].

Under electrocatalytic conditions, scaling relations are further modified by adsorbate–solvent interactions. Directional stabilization arising from hydrogen bonding and electrostatic interactions selectively affects polar intermediates, leading to systematic changes in both slopes and offsets of scaling relations when compared to vacuum calculations [273]. As a consequence, scaling relations derived in the absence of solvent may be shifted or altered in solution, even when the underlying bonding mechanism at the surface remains unchanged.

Interactions with electrolyte species provide an additional source of systematic modification of adsorption energetics in electrochemical environments. Cations at the electrified interface can selectively stabilize or destabilize reaction intermediates in a manner that depends on both the nature of the adsorbate and the metal surface, thereby reshaping adsorption‐energy scaling relations [274]. These adsorbate–electrolyte interactions alter relative adsorption energies in a predictable way, implying that scaling relations established under vacuum conditions may require explicit environmental corrections when applied to electrocatalytic screening.

Nevertheless, despite their limitations, scaling relations provide an invaluable tool for capturing reactivity trends. They also underpin the use of a limited set of descriptors to model more complex processes, and form the foundational concept for further ML approaches based on reaction descriptors.

### 6.2 Reaction Descriptors

**FIGURE 12:**
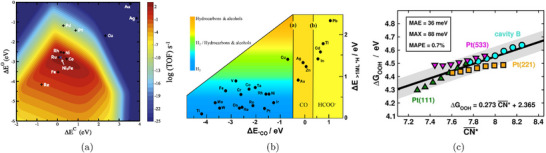
Descriptors of catalytic reactions. (a) Theoretical volcano plot for the production of methane from syngas. The turnover frequency (TOF) is plotted as a heatmap depending on the carbon and oxygen binding energies. Selected catalyst surfaces are marked by the scattered dots. Reproduced with permission [275]. Copyright 2011, CC BY. (b) Descriptors of metal catalyst activity for HER and ${\mathrm{CO}}_{2}\mathrm{RR}$. Relative rates of CO_2_RR and HER are plotted as a function of CO adsorption energy (horizontal axis) and the adsorption energy of hydrogen on top sites (vertical axis) at coverages above one monolayer. Reproduced with permission [111]. Copyright 2018, American Chemical Society. (c) Adsorption energy of the *OOH adsorbate versus the generalized coordination number ${\bar{CN}}^{*}$ for different catalyst surfaces. Reproduced with permission [278]. Copyright 2018, Wiley‐VCH Verlag GmbH & Co.

As most (electro)catalytic processes are governed by a rate‐limiting step (see Section 5.5), the combination of adsorbate energies and TS scaling relations provides a powerful framework to reduce the number of independent variables needed to describe reactivity. As a result, many catalytic systems can be effectively characterized using just one or a few descriptors. When catalytic activity is plotted as a function of these descriptors, it often exhibits a volcano‐shaped trend, reflecting the Sabatier principle: optimal performance occurs when intermediates bind to the surface neither too strongly nor too weakly. An example is shown in Figure 12a, where the activity of ${\mathrm{CH}}_{4}$ formation from syngas, expressed as reaction turnover frequency (TOF), is plotted against the binding energies of carbon and oxygen atoms on the catalyst [275]. Additionally, catalyst selectivity can also be expressed in terms of descriptors, such as the *CO and *H adsorption energies for electrochemical ${\mathrm{CO}}_{2}\mathrm{RR}$ [111]. The adsorption energies of *CO and *H help classify electrocatalysts according to their preference to produce $H_{2}$ or to reduce ${\mathrm{CO}}_{2}$ to CO, ${\mathrm{HCOO}}^{-}$ or more complex hydrocarbons, Figure 12b.

As a result, adsorption energies of key intermediates, or their combinations, are frequently used as reactivity descriptors. Alongside these covalent descriptors, electrostatic properties such as the work function and the PZC have been identified as important factors in electrochemical systems, where the structure of the electric double layer can substantially influence reaction kinetics [276, 277].

Because adsorption energies and local geometric features strongly influence reactivity, only a subset of surface sites typically dominates catalytic activity. This phenomenon, known as structure sensitivity, results in a distribution of site‐specific activities driven primarily by variations in adsorption strength. To efficiently capture these effects without computing each site explicitly, a variety of physical and chemical descriptors have been proposed that correlate with adsorption energies. These include quantities related to the electronic structure, local coordination environment, and thermodynamic stability. By mapping adsorption properties onto such descriptors, it becomes possible to reduce the computational cost associated with modeling complex catalytic surfaces.

One commonly used descriptor for adsorption on transition metal surfaces is the center of the d‐band [279, 280]. In the d‐band model, the adsorbate–metal interaction is divided into contributions from sp and d electrons. The broad sp‐bands contribute a relatively constant baseline to the bond strength due to their similar characteristics across transition metals. Variations in adsorption strength are primarily governed by the narrower and more localized d‐bands. While the d‐band center captures key trends, it has known limitations, especially when comparing distinct surface terminations [281]. The number of valence electrons has also been used to correlate with adsorption energies on both metals and metal oxides [282].

In addition to electronic descriptors, geometric properties have proven effective in predicting adsorption trends. One example is the generalized coordination number, first introduced for pure metals [278, 283] and then extended to alloys [284], which expands on the standard coordination number by accounting not only for the number of nearest neighbors but also for their local environments. For an atom i with $n_{i}$ nearest neighbors, the generalized coordination number is defined as

(63) $${\bar{CN}}^{*}\left(i\right)=\frac{1}{cn_{\max}}\sum_{j}^{n_{i}}\sum_{k}^{n_{j}}\frac{d_{jk}^{\mathrm{bulk}}}{d_{jk}}$$

where $cn_{\max}$ is the bulk coordination of atom i and $d_{jk}^{\mathrm{bulk}}$ and $d_{jk}$ are the distances between the neighboring atoms j and k in the pristine bulk and in the studied system, respectively. If the distances between atoms j and k can be assumed identical to the corresponding bulk values, Equation (63) simplifies to

(64) $${\bar{CN}}^{*}\left(i\right)=\bar{CN}\left(i\right)=\frac{1}{cn_{\max}}\sum_{j}^{n_{i}}n_{j}$$

where $n_{j}$ is the number of atoms coordinated to the j‐th neighbor of atom i.

This purely geometric quantity has been shown to accurately predict adsorption energies across a wide range of surface structures and particle sizes, without requiring explicit electronic structure calculations. The correlation between the adsorption free energies of the *OOH intermediate in the ORR and ${\bar{CN}}^{*}$ is shown in Figure 12c. Here, the adsorption energies are computed on Pt(111), Pt(533), Pt(221), and defected Pt(111) with a cavity. Different points correspond to different values of applied strain [278].

Additionally, the generalized coordination number correlates with electronic properties such as d‐band centers, band fillings, and electrochemical parameters like half‐wave potentials, linking local geometry to reactivity in a simple and transferable way.

While the descriptors discussed above rely on single adsorption energies or individual reaction steps, an emerging class of descriptors aims at capturing the properties of the entire reaction free‐energy landscape. These so‐called reaction‐landscape descriptors incorporate information from multiple elementary steps, or even from the full catalytic cycle, and therefore go beyond traditional volcano‐plot approaches based on one key intermediate or a single potential‐determining step.

**FIGURE 13:**
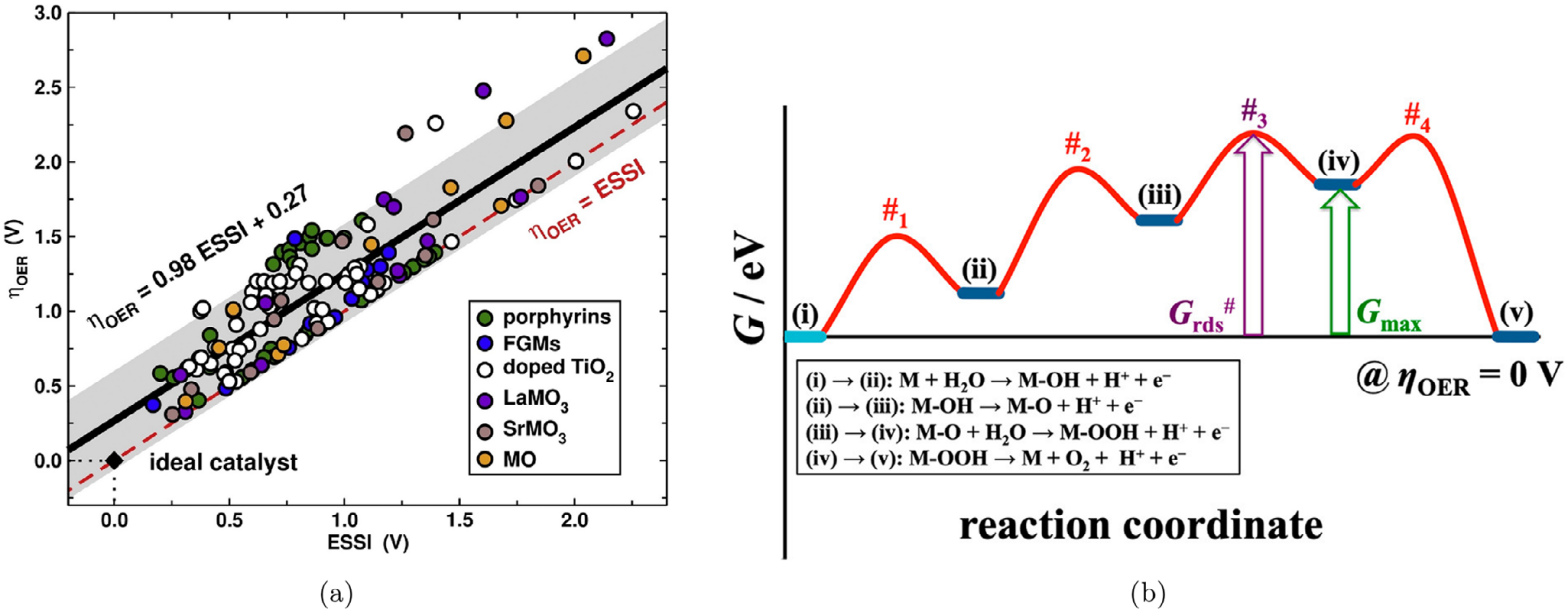
Reaction‐landscape descriptors of catalytic reactions. (a) Theoretical overpotential $\eta_{\mathrm{OER}}$ of the oxygen evolution reaction (OER) plotted as a function of the electrochemical‐step symmetry index (ESSI). The solid black line represents a linear fit to the computed materials, while the red dashed line indicates the condition $\eta_{\mathrm{OER}}=\mathrm{ESSI}$. The ideal catalyst corresponds to ESSI=0 and lies on the red dashed line. Reproduced with permission [285]. Copyright 2018, Elsevier B.V. (b) Free energy steps, free energy of the rate‐determining step $G_{\mathrm{rds}}^{\#}$ (purple arrow) and $G_{\max}$ (green arrow) at zero applied overpotential. Reproduced with permission [286]. Copyright 2020, American Chemical Society.

A representative example is the electrochemical‐step symmetry index (ESSI) [285], which quantifies the degree of energetic asymmetry among all electrochemical steps of a catalytic cycle at the equilibrium potential

(65) $$\text{ESSI}=\frac{1}{n}\sum_{i=1}^{n}\left(\frac{\Delta G_{i}^{+}}{e}-U^{0}\right)$$

where $\Delta G_{i}^{+}$ are the free energy variations along the reaction pathway that are equal or larger than the thermodynamic equilibrium potential $U^{0}$ and n is the number of steps with $\Delta G_{i}\geq eU^{0}$. In general, ESSI≤η, the thermodynamic overpotential, and the ideal catalyst shows ESSI=0, see Figure 13a. Rather than focusing on a single limiting step, ESSI accounts for the cumulative imbalance of reaction free energies across the pathway, providing a descriptor that reflects how evenly the overall reaction driving force is distributed. This formulation has been shown to rationalize experimental activity trends for oxygen evolution catalysts more reliably than descriptors based solely on the thermodynamic overpotential [287].

A further generalization is provided by the descriptor $G_{\max}\left(\eta \right)$, which is rooted in the free‐energy span model originally developed for homogeneous catalysis and subsequently adapted to electrocatalytic reactions [286]. At a given applied overpotential η, $G_{\max}\left(\eta \right)$ corresponds to the largest free‐energy difference between the highest‐ and lowest‐lying intermediates along the reaction pathway, thereby defining an upper bound on the effective kinetic bottleneck of the catalytic cycle, see Figure 13b. Unlike equilibrium‐based descriptors, $G_{\max}\left(\eta \right)$ explicitly accounts for the effect of the applied potential and does not rely on the assumption that the potential‐determining and rate‐determining steps coincide. As a result, it enables a consistent ranking of catalysts across a wide potential range and has been shown to outperform single‐step descriptors in reproducing experimental activity trends for multi‐electron electrocatalytic reactions [288].

Comprehensive discussions of the theoretical foundations, definitions, and practical use of reaction‐landscape descriptors, including ESSI, $G_{\max}\left(\eta \right)$, and their relation to different generations of volcano plots, can be found in recent dedicated reviews [289, 290].

### 6.3 ML Algorithms for Electrocatalyst Screening

Reaction descriptors such as adsorption energies can be computed with good accuracy using DFT. However, direct screening of large numbers of candidate materials through quantum mechanical calculations remains computationally demanding, posing a challenge for high‐throughput catalyst discovery. ML has emerged as a powerful approach to analyze existing data, uncover underlying correlations, and identify new promising materials with high predictive accuracy and low computational cost. This data‐driven strategy is not only capable of predicting adsorption energies and other relevant properties, but also of providing mechanistic insight into catalytic activity and selectivity. For more comprehensive overviews of ML methods and their application to electrocatalysis, the reader is referred to recent reviews on this topic [291, 292, 293, 294].

**FIGURE 14:**
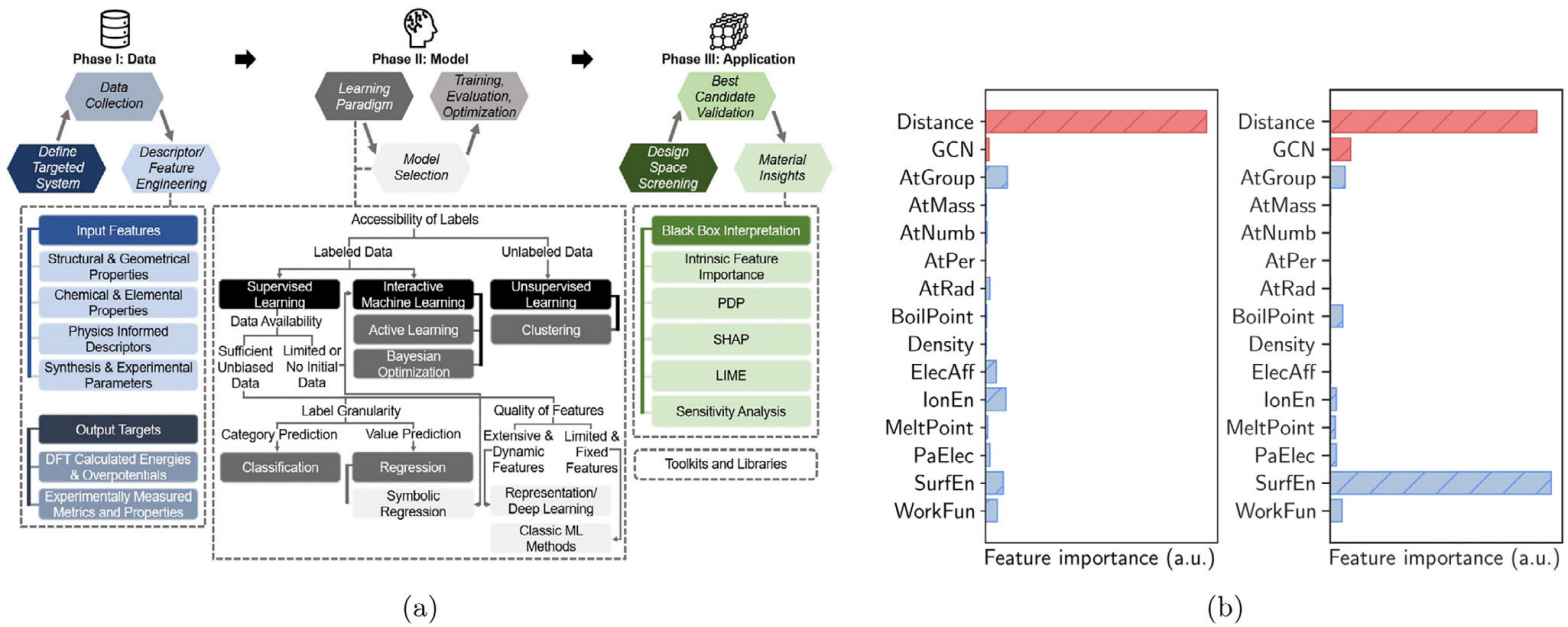
ML models for catalyst discovery. (a) Diagram of the three‐phase pipeline (data, model, application) for applying ML techniques to catalyst design. Reproduced with permission [293]. Copyright 2024, The Royal Society of Chemistry. (b) Importance of the input features for classification (left panel) and regression (right panel) models of CO adsorption on low‐concentration Cu‐based bimetallic alloys. Geometrical and chemical features are reported in orange and light blue, respectively. Reproduced under the terms of the CC‐BY 3.0 license [296]. Copyright 2024, The Authors, published by the Royal Society of Chemistry.

As shown in Figure 14a, the standard ML pipeline for electrocatalyst screening typically consists of three main stages: data preparation and representation, model development, and validation, and model application [293]. The first stage involves collecting data either from experiments or from DFT calculations, and assigning descriptive features to the selected atomic structures. These input features aim to capture the relevant physical and chemical properties of the systems under study. Common features include coordination numbers, atomic numbers, electronegativity, and local electronic structure descriptors, such as d‐band center or work function.

In the second stage, ML models are trained to map input features to target properties such as adsorption energies or activation barriers. A wide range of ML algorithms have been used in this context, including linear regression, decision trees, kernel methods, and neural networks. The performance of a model depends strongly on the choice of features, the quality/amount of the training data, and the optimization of hyperparameters. To avoid overfitting and ensure robustness, the training process is typically accompanied by validation against a held‐out subset of the data. Automated tools such as TPOT [295] can assist in model selection and optimization.

The third stage of the pipeline involves using the trained model to explore new chemical space, screen hypothetical materials, or guide the selection of catalyst candidates. These applications allow for rapid prediction of target properties without additional DFT calculations, significantly accelerating the discovery process.

This general framework has been applied successfully to a range of electrocatalytic problems. In one example, CO adsorption energies on intermetallic surfaces were predicted using an active ML approach [297, 298]. Adsorption sites were described by a set of physical and chemical fingerprints that included atomic number, Pauling electronegativity, coordination number with the adsorbate, and the median adsorption energy of the corresponding pure element, allowing both steric and electronic effects to be captured. Regression models trained on these fingerprints were used to identify bimetallic surfaces with near‐optimal adsorption energies for CO and H in the context of ${\mathrm{CO}}_{2}\mathrm{RR}$ and HER, with predictions confirmed experimentally.

A related approach employed a combination of classification and regression to predict CO adsorption on copper‐based bimetallic alloys [296]. The classification model was first used to identify stable adsorption sites, and regression was then applied to estimate their corresponding adsorption energies. To gain insight into the physical factors driving these predictions, the authors performed a feature importance analysis, a method used to assess which input features most strongly influence the model's predictions. As shown in Figure 14b, the distance between the CO adsorbate and the alloyed metal atom emerged as the most influential feature in both classification and regression tasks, underscoring its key role in determining adsorption behavior. The regression model also assigned high importance to the surface energy of the pristine guest metal species, suggesting that the CO adsorption energy effectively reflects the intrinsic reactivity of heteroatom surfaces. This approach was subsequently extended to the screening of copper‐based bimetallic alloys for CO–CO dimerization, revealing that the incorporation of p‐block elements, such as Al and Ga, leads to a systematic reduction of the dimerization activation barrier [299].

Other studies have employed ML to map CO binding energies on bimetallic surfaces [300], enabling rapid screening of active facets and revealing isolated Ni sites surrounded by surface Ga atoms that favor on‐top CO adsorption. These motifs deviate from the typical bridge or three‐fold hollow configurations on pure Ni and exhibit step‐like kinetic behavior even on close‐packed facets, providing thermodynamically favorable sites for CO reduction.

These examples illustrate how ML models trained on physically meaningful descriptors can significantly reduce the computational cost of catalyst screening, while retaining predictive accuracy sufficient to identify active and selective sites. As these models become increasingly refined, they not only improve the efficiency of property prediction but also contribute to a deeper understanding of structure–property relationships across diverse chemical systems. However, the applicability of such approaches is often constrained by the availability of reliable training data and by the assumption that reactions proceed on static PES. To enable the simulation of dynamical effects over longer timescales and larger systems, recent efforts have focused on the development of ML force fields for MD simulations.

### 6.4 ML Force Fields

As anticipated in Section 3.3, the explicit inclusion of both solvent and electrolyte in MD simulations provides the most comprehensive framework for capturing the full complexity of the electrochemical interface. However, AIMD simulations are typically restricted to relatively small systems (up to a few hundred atoms) and short timescales (tens of picoseconds) due to their computational cost. This leads to unavoidable finite‐size effects, difficulties in achieving proper equilibration, and insufficient statistical sampling. Moreover, certain processes remain entirely inaccessible on the timescale of typical AIMD simulations. For example, the diffusion of water molecules between the interfacial and bulk regions of the solvent has been reported to occur on a timescale of approximately 500 ps [301]. To reach nanosecond timescales and simulate larger systems, the scalability of force fields (FFs) becomes essential.

**FIGURE 15:**
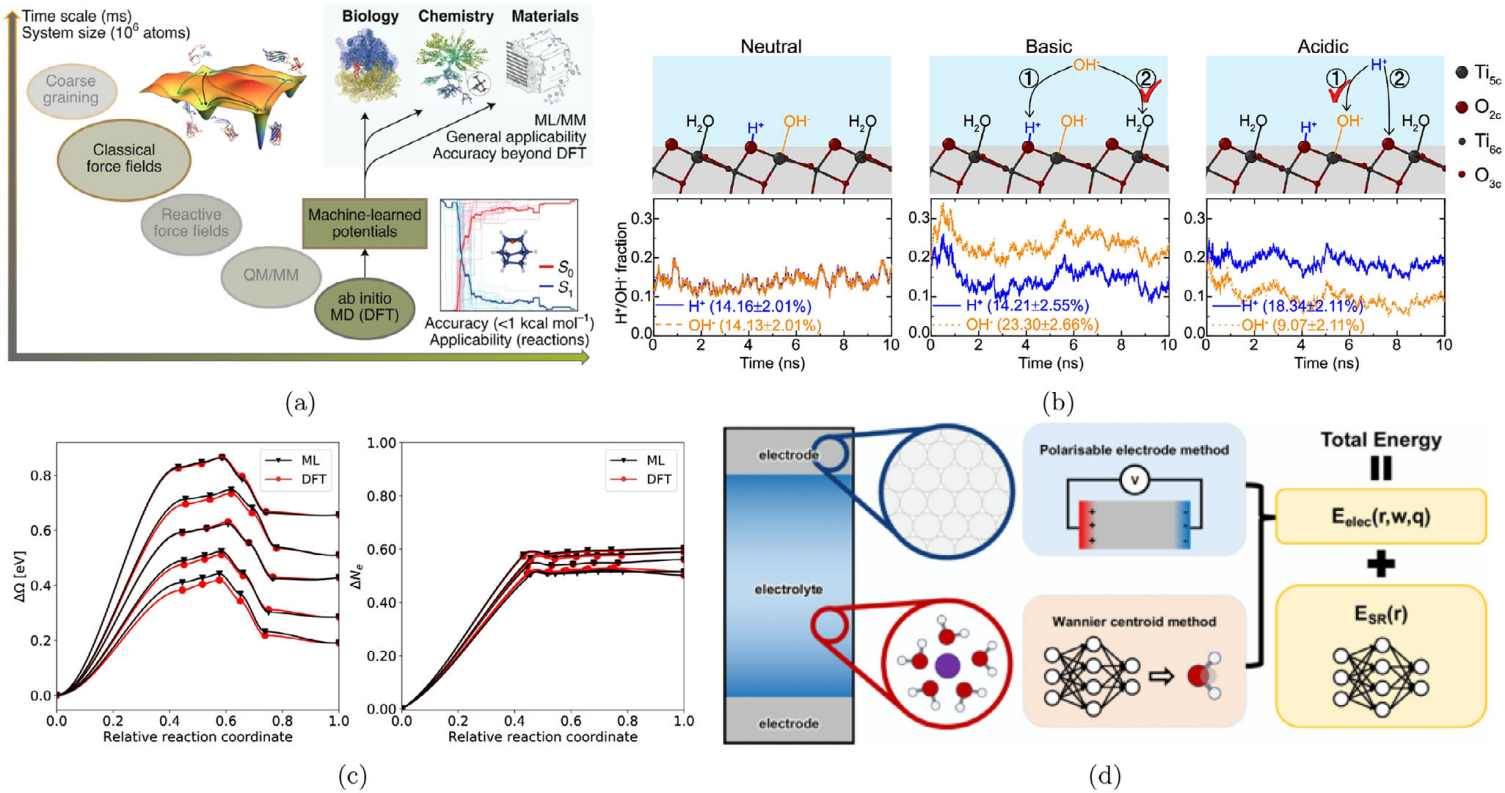
ML‐FFs applied to electrochemical interfaces. (a) Accuracy/applicability to reactions vs attainable timescale/system size of different MD techniques, including AIMD, classical FFs and ML‐FFs. The insets show possible applications of ML‐FFs. Reproduced with permission [304]. Copyright 2021, Springer Nature Limited. (b) Time evolution of surface H^+^ and OH^−^ coverages on TiO_2_ interfaces in (left panel) neutral, (central panel) basic, and (right panel) acidic solutions. The central and right panels illustrate possible adsorption pathways of additional solvated water ions. Reproduced under the terms of the CC‐BY‐NC‐ND 4.0 license [321]. Copyright 2024, The Authors, published by Springer Nature. (c) Comparison of the reaction pathway (left panel) and charge transfer (right panel) along the reaction coordinate for the Volmer step of HER, obtained from standard DFT‐based NEB calculations and ML‐assisted NEB, at different electrode work functions ranging from 4.0 to 4.8 V (from bottom to top). Reproduced under the terms of the CC‐BY 4.0 license [322]. Copyright 2023, The Authors, published by Springer Nature. (d) Scheme of an ML‐FF including electrostatic interactions and dielectric response through a hybrid approach based on Wannier centroids of H_2_O molecules and a classical Siepmann‐Sprik model of the metal electrode. Reproduced with permission [323]. Copyright 2025, American Physical Society.

Classical FFs rely on fixed, hand‐crafted functional forms that are fitted to reproduce selected properties from experiments or quantum mechanical calculations. This design inherently limits their flexibility and transferability, often resulting in insufficient accuracy for electrochemical systems. In contrast, machine‐learning force fields (ML‐FFs), also known as machine‐learning interatomic potentials, offer much greater flexibility through the large numbers of trainable parameters and can be fitted directly to forces computed from ab initio methods [302, 303]. As such, ML‐FFs promise to combine the accuracy of ab initio methods with an efficiency and scalability comparable to the classical ones. The advantages of ML‐FFs are summarized in Figure 15a, where the trend in accuracy for reactive events versus system size/timescale accessible for different MD techniques is reported: ML‐FFs can merge together the best of the classical and ab initio MD worlds [304].

An ML‐FF is typically trained on a dataset of atomistic configurations, from which it learns to predict total energies, atomic forces, and, when required, other quantities such as virials, atomic charges [305], Wannier centroids [306], or atomic electronegativities [307]. The generation of the dataset is often the most critical step, as ML models generally interpolate well within the training data but may fail when extrapolating beyond it [308]. Therefore, the dataset must cover all relevant regions of the PES while being constructed efficiently to avoid unnecessary and expensive ab initio calculations. It is worth remarking the importance of data‐efficiency in the creation of the dataset since ML‐FFs have been raised as an alternative to AIMD and the computational cost required for generating the dataset should not overcome the cost of the original fully ab initio approach. For this reason, many strategies for efficient and exhaustive dataset generation have been proposed [309, 310, 311].

This review does not attempt to cover all ML models used to fit the PES. For more comprehensive discussions, the reader is referred to existing reviews [302, 312]. Instead, attention is drawn here to a few essential characteristics shared by most ML‐FFs, which may guide users through the growing landscape of available models.

In general, ML‐FFs are local by construction. An ML model predicts properties such as local energy contributions or atomic forces based only on the atomic configuration within a finite cutoff radius (typically around 6 Å). Global properties, such as the total energy, are then computed as the sum of contributions from local environments. More advanced approaches have been developed to partially extend the receptive field beyond the nominal cutoff, for instance using message‐passing techniques [313].

To overcome the limitations associated with short‐range locality, a variety of schemes have been proposed to explicitly include long‐range interactions, such as dispersion forces, electrostatics, and induction effects [307, 314]. Although long‐range models represent a very active area of research [315, 316, 317], they have mainly been tested on relatively simple systems and are not yet commonly applied to more complex conditions.

Another emerging focus in the ML‐FF community is the incorporation of NQEs [302]. These effects can be properly accounted for using Path‐Integral Molecular Dynamics (PIMD), which relies on the isomorphism between a quantum particle and a ring polymer composed of P harmonically coupled classical replicas [130]. Because PIMD simulations require propagating P replicas of the system (typically with P between 16 and 32), the computational cost of fully ab initio PIMD becomes prohibitive for complex systems. ML‐FFs provide a tractable alternative to replace the ab initio engine in PIMD, enabling simulations that include NQEs in systems where the dynamics of light atoms, such as protons, is essential. Electrocatalytic interfaces are a clear example of this, given the central role played by hydrogen‐related processes.

#### 6.4.1 Recent Development of ML‐FFs in Electrocatalysis

The high computational cost associated with AIMD simulations of electrocatalytically relevant interfaces has long motivated the application of ML‐FFs to these systems [301, 318, 319, 320]. One of the earliest studies in this area examined low‐index Cu surfaces in contact with liquid water [319], providing a detailed analysis of structural and dynamical properties, emphasizing the impact of system size on the results. Thanks to their scalability, ML‐FFs enable the quantification of finite‐size effects that are otherwise inaccessible in AIMD. Surface complexity was increased in subsequent works [320] by considering stepped Cu facets, revealing distinct solvent behavior near step edges compared to flat terraces. ML‐FFs also allow the investigation of dynamical processes at the metal–solvent interface that lie beyond the timescale of AIMD. For instance, interfacial water dynamics on Pt(111) involves rapid diffusion within the surface layers on a timescale of approximately 30 ps and much slower exchange with the bulk, occurring in around 500 ps [301].

Initial studies focused on interfaces with pure water, but more recent work has extended this framework to more realistic electrolytes. In one example, ${\mathrm{Na}}^{+}$ and ${\mathrm{Cl}}^{-}$ ions were introduced into water in contact with a TiO_2_ surface [321]. Using an ML‐FF, the structure of the EDL was revisited from an atomistic perspective, revealing more complex ionic layering in the Stern region. The effect of pH on surface charge and ionic rearrangement was also characterized, as shown in Figure 15b. Solving a 1D Poisson equation using Gaussian charge distributions centered on atomic positions and the average Wannier centroids of water molecules, the potential drop across the interface was quantified under varying conditions. The results are employed in a linearized capacitance model that qualitatively reproduces the pH dependence of the EDL.

Reactivity at the catalyst–solvent interface is another area where ML‐FFs have proven valuable, particularly when coupled with enhanced sampling techniques (see Section 5.3). One study employed ML‐accelerated MD with umbrella sampling to compute reaction barriers for HER on Pt(111) in the presence of ${\mathrm{Cl}}^{-}$ [233]. The results showed that the Tafel step is favored at high hydrogen coverage, while the Heyrovsky step dominates at low coverage. In another study, the same system was examined with a focus on NQEs [324]. Combining Path Integral and constrained Monte Carlo simulations, accelerated by ML‐FFs, the authors demonstrated that NQEs reduce the activation barriers for the Volmer and Heyrovsky steps of HER by approximately 0.1 eV. The Tafel step remained unaffected, suggesting that the inclusion of NQEs can shift the preferred reaction pathway.

While HER offers a relatively simple test case, other investigations have targeted more complex adsorbates and reaction mechanisms. For example, some studies have addressed the behavior of intermediates such as *CO, *OH, *COH, *CHO, and *OCCHO on Cu and Ag surfaces in explicit solvent environments [325, 326]. Structural rearrangements of the catalyst surface under reactive conditions, such as CO‐induced reconstruction, have also been examined [327]. Although these efforts contribute valuable insights into realistic electrocatalytic processes [233, 325, 328], a major limitation remains the lack of a consistent framework for including the applied potential within ML‐FF‐based simulations. As a result, the driving force of electrocatalytic transformations is often poorly represented.

To address this issue, one study implemented a grand canonical formalism to model HER on Au(111) [322]. In this approach, ML‐FFs were not used for molecular dynamics but to accelerate NEB calculations (see Section 5.2). The ML model was trained to reproduce the grand canonical energy, expressed as a Taylor expansion in the charge, and the dataset was generated using GC‐DFT with an implicit solvent and a few explicit water molecules. This strategy produced a charge‐aware ML model capable of capturing potential‐dependent energetics under constant‐potential conditions. The effectiveness of this model is shown in Figure 15c, in which the ML‐FF is able to reproduce the reaction pathway and the charge transfer for the Volmer step of HER, as predicted by DFT NEB calculations, at different values of applied potential.

More recently, another approach was developed to incorporate the influence of the electric field into ML‐FF simulations [323]. A hybrid scheme, shown in Figure 15d, was introduced to simultaneously describe the dielectric response of both the metal electrode and the electrolyte. In this model, electronic polarization in the electrolyte is inferred from average Wannier centroids predicted by an ML model, while the nonlocal charge transfer within the metallic electrode is treated classically via the Siepmann‐Sprik model [329]. A constant‐charge formalism was implemented, fixing the number of ions in the electrolyte and performing simulations at potentials above and below the PZC. By applying a small external field, the dielectric response was also characterized, and the origin of the observed negative differential capacitance was attributed to the presence of chemisorbed water molecules.

Building on these initial efforts to incorporate surface electrification into ML‐FFs, a series of recent independent studies proposed diverse strategies to tackle this challenge [330, 331, 332, 333]. For instance, ref. [330] introduced a potential‐aware ML‐FF architecture operating within a constant‐charge framework. They subsequently applied a Legendre transformation to the electronically canonical free energies (see Section 4.3) to enable analysis in a constant‐potential ensemble, determining the preferential adsorption charge of OH on Cu(100) under varying applied potentials and pH conditions. In contrast, ref. [331] focused on the development of a highly data‐efficient ML‐FF architecture designed to minimize the number of configurations required in the training dataset. This approach facilitates advanced, computationally demanding simulation setups, such as those employing accurate meta‐GGA functionals for water‐network description and the rigorous double‐reference method for modeling surface electrification.

In summary, ML‐FFs have enabled significant progress in the atomistic modeling of electrocatalytic interfaces, allowing simulations that were previously out of reach in terms of system size, timescale, and complexity. These methods have proven effective not only for structural and dynamical studies but also for investigating reactivity and quantum effects in realistic environments. At the same time, important challenges remain, particularly regarding the consistent inclusion of applied potential and long‐range interactions. The field is developing rapidly, with ongoing efforts addressing current limitations and proposing new frameworks. As models improve, further advances in accuracy, transferability, and predictive power can be expected. This area of research remains highly active and is likely to produce substantial methodological and practical breakthroughs in the near future.

## 7 Perspectives

The methodological frameworks outlined in this review reflect the progressive formalization of computational strategies in electrocatalysis. These strategies are rooted in DFT and increasingly structured in layers of physical refinement, each aimed at resolving specific features of the electrochemical interface. The study of reaction thermodynamics, the modeling of solvation, the inclusion of electrode potential, and the explicit calculation of kinetic barriers all reflect different levels of abstraction and approximation. What emerges is not a single unified method, but a set of modular approaches that can be combined according to the physical regime and the modeling objective. In practice, these methods are selected based on the quantity of interest, the complexity of the system, and the available computational resources. Table 1 provides a comparative overview of the main strategies discussed in the review, summarizing their objectives, strengths, and known limitations. This synthesis is intended to support informed choices by researchers aiming to model electrocatalytic systems with a balance between realism, cost, and interpretability.

**TABLE 1:** Summary table of the computational methods in electrocatalysis described in the review, with key advantages, disadvantages and goals.

Modeling goal	Method	Advantages	Disadvantages	Notes
Thermodynamics of simple reactions with small adsorbates with negligible dipole	CHE	Implicit treatment of electrons and protons Neutral‐cell calculations Very low computational load	Missing effects of solvation Missing electric fields No kinetic barriers Only PCET	Section 2
Inclusion of solvation effects	Implicit solvation	Inclusion of electrolyte response Easy enforcement of charge‐neutrality Fermi level alignment for potential control Low computational load	Local interactions approximated or neglected No explicit hydrogen bonding Approximated EDL capacitance	Section 3.2
	Explicit solvation	Local interactions and hydrogen bonds Dynamic response of electrolyte with MD Correct description of the EDL	Complex introduction of explicit counterions Short timescales available with AIMD High computational load	Section 3.3
	QM/MM	Quantum description of local interactions Classical coarse‐grained electrolyte Medium/low computational load	Embedding scheme QM/MM boundary surface Code availability	Section 3.3.1
Inclusion of electrostatics	Constant‐charge	Simple cell setup Compatible with all DFT codes	Finite‐size effects Need for extrapolation schemes Large number of calculations Medium/high computational load	Section 4.1
	Constant‐potential	Consistent with experimental conditions Immediate control of electrode potential	Need for GC‐DFT calculation setups Careful Fermi level alignment Slow convergence	Section 4.2
Kinetic barriers and reaction rates	NEB and dimer methods	Compatible with solvation Compatible with explicit bias Identification of charge‐transfer TS	No dynamical electrolyte effects Slow convergence Medium/high computational load	Section 5.2
	Enhanced sampling in MD	Full exploration of the FES Dynamic electrolyte effects	Careful choice of CVs High computational load	Section 5.3
High‐throughput catalyst screening	DFT + ML algorithms	Exploitation of reaction descriptors Exploitation of scaling relations Large chemical space exploration Low computational load of ML algorithms	Large dataset building Careful choice of features and descriptors Detailed benchmarking of predictions	Section 6.3
Full dynamical effects and EDL description	AIMD	Fully ab initio description of the electrolyte Quantum‐mechanical description of interactions Inclusion of applied bias	Short attainable time and spatial scales Reduced number of atoms Careful choice of functional High computational load	Section 3.3
	ML‐FF	Near ab initio accuracy Large time and spatial scales Low computational cost of trajectories	Large training dataset needed Careful choice of functional Careful choice of ML architecture Inclusion of long‐range interactions Careful dataset building	Section 6.4

The structure of available methods is increasingly modular and many of these techniques have been tested extensively on specific systems. Nevertheless, a comprehensive general framework for evaluating their robustness across diverse chemical environments and operating conditions is still missing. Further progress will likely depend not just on the invention of new methods, but on a deeper understanding of how existing ones behave across different length scales, electrochemical regimes, surface structures and how effectively they can be combined.

A major area of consensus is the inadequacy of solvation and EDL modeling in current practice. Implicit solvent models are computationally efficient and provide a useful first approximation, but they fail to capture key features such as ion‐specific effects, pH dependence, and solvent reorganization. Hybrid approaches, combining explicit water layers with continuum embedding and electrostatic boundary conditions, offer a promising path forward. These methods can capture cation‐induced stabilization, competitive adsorption, and field effects that are otherwise inaccessible. Integrating hybrid solvation schemes into simpler workflows is increasingly recognized as essential for realistic electrochemical modeling.

Similarly, modeling electrochemical bias remains a frontier of development. Explicitly controlling the potential in AIMD, rather than relying on fixed‐charge trajectories, will be essential to move beyond idealized interfacial dynamics and access realistic electrocatalytic environments. In parallel, ML and data‐driven models are opening new dimensions for accelerated discovery. In particular, ML‐FFs trained on DFT data now enable enhanced sampling of large, solvated systems at near ab initio accuracy. Active learning frameworks and surrogate models are emerging to explore reaction networks, solvation energies, and bias‐dependent energetics at scale. However, current ML models often rely on limited training sets and lack robust error control. The wider adoption of ML in electrochemical modeling will depend on the development of training protocols that reflect electrochemical conditions (e.g., potential control) and validation against key observables such as reaction onset potentials, differential capacitance, or Tafel slopes.

At the core of these efforts lies the fundamental task of quantifying uncertainty in atomistic simulations. Without standardized protocols, simulations risk producing plausible results from invalid assumptions. While the qualitative insights provided by DFT are often robust, quantitative predictions are typically affected by systematic errors (from functional choice, supercell size, solvation treatment, or thermostats) that are rarely assessed in a principled way. This complicates the comparison between different simulations and weakens the connection with experiments. One of the most important goals for future work is therefore the construction of modeling protocols that allow for controlled approximations, transparent reporting of assumptions, and estimation of uncertainty in computed observables.

More broadly, the challenge is to connect atomistic simulations to experimentally measurable quantities in a systematic and physically meaningful way. Many of the quantities of interest in electrocatalysis, such as activity trends, selectivity ratios, or potential‐dependent current densities, are influenced by phenomena occurring across multiple scales. Connecting the electronic structure of active sites and the observable behavior of an electrochemical cell requires a combination of quantum mechanics, statistical mechanics, and continuum theory. It also requires simulations to be designed with experimental comparability in mind, taking into account how quantities such as work function, interfacial charge, or proton activity relate to the reference electrodes and operating conditions used in practice. The development of shared benchmarks and validation targets, derived from well‐characterized systems under controlled conditions, would help align theoretical predictions with experimental reality. In parallel, more systematic efforts are needed to define what constitutes a meaningful level of agreement between theory and experiment in this context.

Finally, future progress in this field will most probably depend not only on new methods, but on the integration of existing ones into coherent and reproducible workflows. These workflows must be capable of combining electronic structure calculations with sampling methods, solvation models, potential control schemes, and kinetic formalisms in a way that is physically consistent and computationally feasible. Achieving this integration will require technical infrastructure, including standardized input formats, interoperable simulation codes, and accessible repositories for sharing models and datasets. It will also require conceptual clarity about how different levels of theory relate to each other and what assumptions are being introduced when they are coupled. The final aim is the construction of theoretical tools that can provide insight into electrochemical phenomena through clear, testable, and physically grounded mechanisms. The methods discussed in this review form the basis for that goal, and their continued development and application represent the foundation of future progress in computational electrocatalysis.

## Conflicts of Interest

The authors declare no conflicts of interest.

## Data Availability

The authors have nothing to report.
